# Essential Role for the Phosphatidylinositol 3,5-Bisphosphate Synthesis Complex in Caspofungin Tolerance and Virulence in *Candida glabrata*

**DOI:** 10.1128/AAC.00886-19

**Published:** 2019-07-25

**Authors:** Deepak Kumar Choudhary, Priyanka Bhakt, Rupinder Kaur

**Affiliations:** a Laboratory of Fungal Pathogenesis, Centre for DNA Fingerprinting and Diagnostics, Hyderabad, Telangana State, India; b Graduate Studies, Manipal Academy of Higher Education, Manipal, Karnataka, India

**Keywords:** phosphatidylinositol 3,5-bisphosphate signaling, PI(3,5)P2 phosphatase Fig4, intracellular survival, cell wall chitin, biofilm formation, metal ion tolerance, vacuole morphology

## Abstract

Increasing resistance of the human opportunistic fungal pathogen Candida glabrata toward the echinocandin antifungals, which target the cell wall, is a matter of grave clinical concern. Echinocandin resistance in C. glabrata has primarily been associated with mutations in the β-glucan synthase-encoding genes C. glabrata *FKS1* (*CgFKS1*) and *CgFKS2*. This notwithstanding, the role of the phosphoinositide signaling in antifungal resistance is just beginning to be deciphered. The phosphatidylinositol 3,5-bisphosphate [PI(3,5)P2] is a low-abundance lipid molecule that is pivotal to the intracellular membrane traffic. Here, we demonstrate for the first time that the PI(3,5)P2 kinase CgFab1, along with its activity regulator CgVac7 and the scaffolding protein CgVac14, is required for maintenance of the cell wall chitin content, survival of the cell wall, and caspofungin stress. Further, deletion analyses implicated the PI(3,5)P2 phosphatase CgFig4 in the regulation of PI(3,5)P2 levels and azole and echinocandin tolerance through CgVac14. We also show the localization of the CgFab1 lipid kinase to the vacuole to be independent of the CgVac7, CgVac14, and CgFig4 proteins. Lastly, our data demonstrate an essential requirement for PI(3,5)P2 signaling components, CgFab1, CgVac7, and CgVac14, in the intracellular survival and virulence in C. glabrata. Altogether, our data have yielded key insights into the functions and metabolism of PI(3,5)P2 lipid in the pathogenic yeast C. glabrata. In addition, our data highlight that CgVac7, whose homologs are absent in higher eukaryotes, may represent a promising target for antifungal therapy.

## Abstract (precis)

Increasing resistance of the human opportunistic fungal pathogen Candida glabrata toward the echinocandin antifungals, which target the cell wall, is a matter of grave clinical concern. Echinocandin resistance in C. glabrata has primarily been associated with mutations in the β-glucan synthase-encoding genes C. glabrata *FKS1* (*CgFKS1*) and *CgFKS2*. This notwithstanding, the role of the phosphoinositide signaling in antifungal resistance is just beginning to be deciphered.

## INTRODUCTION

Candida glabrata is a human opportunistic fungal pathogen of high clinical significance (1–3). It is the second to fourth most common cause of *Candida* bloodstream infections, based on the geographical area (3–5). C. glabrata is intrinsically less susceptible to widely used azole antifungals, compared to other *Candida* species in the CTG clade, thereby rendering the azole antifungal therapy ineffective (6, 7). An increase in C. glabrata infections in last 2 decades has been associated with the increased use of azoles in some studies (3, 5, 8, 9). Azole antifungals inhibit ergosterol biosynthesis by binding to the iron of the porphyrin ring in the active site of the enzyme lanosterol 14-α-demethylase, which converts lanosterol into 4,4′-dimethyl cholesta-8,14,24-triene-3-β-ol and is encoded by the C. glabrata *ERG11* (*CgERG11*) gene (6).

C. glabrata is also known to acquire resistance to both azole and echinocandin antifungals (1–3, 10, 11). Echinocandins impede cell wall biosynthesis by inhibiting the synthesis of its principal structural component β-1,3-glucan (10). These noncompetitively bind to the catalytic subunits of β‐(1,3)-glucan synthase enzymes, encoded by three genes: *CgFKS1*, *CgFKS2*, and *CgFKS3* (10). Mutations in hot-spot regions of *CgFKS1* and *CgFKS2* genes have frequently been associated with echinocandin resistance (1, 2, 10–12). Contrarily, overexpression of multidrug efflux pumps is the most prevalent mechanism of azole resistance in C. glabrata (7). Multiple factors have been identified that contribute to the cellular response to azole and echinocandin antifungals, underscoring that antifungal tolerance in C. glabrata is a complex process (12–18).

The phosphoinositide signaling pathway regulates various physiological processes, including protein trafficking, autophagy, cell cycle progression, and hyphal morphogenesis (19, 20). Recently, phosphoinositides have also been implicated in antifungal resistance in the pathogenic fungi C. albicans and C. glabrata (17, 21). The phosphatidylinositol-4-phosphate has been shown to be pivotal to trafficking of the major multidrug transporter, Cdr1, to the plasma membrane in C. albicans (21). The phosphatidylinositol-4-kinase Stt4 was found to a genetic interactor of Hsp90 and required for antifungal tolerance in C. albicans (22). Similarly, the phosphatidylinositol 3,5-bisphosphate [PI(3,5)P2] kinase CgFab1 has recently been reported to be essential for actin cytoskeleton reorganization and azole stress survival in C. glabrata (17).

PI(3,5)P2 constitutes a very small fraction of total phosphoinositides in the nonpathogenic yeast Saccharomyces cerevisiae and plays an important role in membrane trafficking and vacuole homoeostasis (23–26). It is predominantly present in the vacuolar membrane (24–26). PI(3,5)P2 synthesis and turnover is modulated by the PI(3,5)P2 regulatory complex, which consists of the PI(3,5)P2 kinase Fab1, the regulatory proteins Vac7 and Vac14, PI(3,5)P2 phosphatase Fig4, and the phosphoinositide-binding protein Atg18 (24, 25). Fab1 converts phosphatidylinositol 3-phosphate to PI(3,5)P2, and its kinase activity is positively and negatively regulated by the Vac7 and Vac14 proteins and the Atg18 protein, respectively (23, 27–30). Consistently, PI(3,5)P2 levels are significantly diminished in *vac7* and *vac14* mutants (27, 28) and elevated in the *atg18* mutant in S. cerevisiae (30).

We have previously shown that CgFab1, CgVac7, and CgVac14, components of the PI(3,5)P2 synthesis complex in C. glabrata, are required for maintenance of vacuole size and azole antifungal tolerance (17). However, the contribution of these components to echinocandin tolerance is not known. Hence, we have extended our analysis of PI(3,5)P2 signaling modulators further and report for the first time that CgVac7 and CgVac14 are pivotal to cell wall and metal ion homeostasis and caspofungin tolerance in C. glabrata. Besides uncovering an essential role for CgFab1, CgVac7, and CgVac14 in biofilm formation, intracellular survival, and virulence, we demonstrate the vacuolar localization of CgFab1 to be independent of CgFig4, CgVac7, and CgVac14 proteins. In addition, we show, through genetic analyses, that the antifungal susceptibility defects of *Cgfab1Δ* and *Cgvac7Δ* mutants, but not of *Cgvac14Δ* mutant, are restored upon disruption of the PI(3,5)P2 phosphatase-encoding gene *CgFIG4*. Lastly, overexpression of the PI(3,5)P2 synthesis complex components revealed a central role for CgFab1 and CgVac7 in rescuing phenotypes associated with CgVac14 loss. Overall, our data unveil for the first time a complex genetic relationship among different constituents of the PI(3,5)P2 regulatory complex in C. glabrata.

## RESULTS

### The PI(3,5)P2 synthesis complex components, CgVac7 and CgVac14, are required for caspofungin tolerance.

To delineate the role of PI(3,5)P2 signaling in antifungal tolerance in C. glabrata, we deleted the *CgFIG4* gene from the genome. The S. cerevisiae ortholog of *CgFIG4* codes for a lipid phosphatase that removes phosphate from position 5 of the inositol ring of PI(3,5)P2 and generates PI3P (29). CgFig4 showed 72% identity with S. cerevisiae Fig4 and contains both SacI homology and phosphoinositide polyphosphatase domains (see Fig. S1A in the supplemental material). Similarly, CgVac7 possessed two vacuolar segregation protein 7 (Vac7) domains (Fig. S1A) and was 37% identical to ScVac7. Of note, Vac7 homologs are absent in higher eukaryotes (26). Further, consistent with the role of ScVac14 as a scaffold for the PI(3,5)P2 regulatory complex (31), CgVac14 also displayed binding regions for both CgFab1 and CgFig4 enzymes (Fig. S1A). The amino acid sequence identities of CgVac14 and CgFab1 with their S. cerevisiae counterparts were 59 and 52%, respectively.

**FIG 1:**
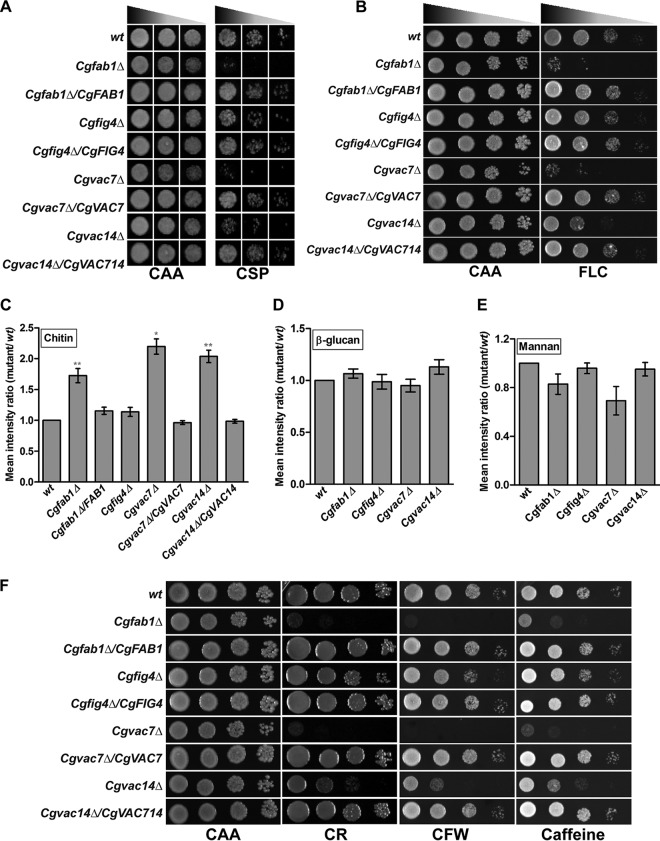
Components of the PI(3,5)P2 synthesis complex are required for antifungal tolerance and cell wall homeostasis. (A) Caspofungin susceptibility of indicated C. glabrata strains, as assessed by liquid growth-based assay. Cultures were inoculated at an initial OD_600_ of 0.2 and grown in medium lacking (CAA) or containing 75 ng/ml caspofungin (CSP) for 24 h, and 3 μl of 25-, 125-, and 625-fold-diluted cultures was spotted on the CAA medium. (B) Fluconazole susceptibility of indicated C. glabrata strains, as assessed by a serial dilution spot assay on the solid medium. Overnight-grown C. glabrata cultures were 10-fold serially diluted, and 3 μl of each dilution was spotted on medium lacking (CAA) or containing 16 μg/ml fluconazole (FLC). (C to E) Flow cytometry-based measurement of chitin (C), β-glucan (D), and mannan (E) after cell staining with 2.5 μg/ml calcofluor white, 12.5 μg/ml aniline blue, and 1 μg/ml concanavalin A, respectively. Data (*n* = 3 to 6) are presented as mean fluorescence intensity ratios ± the standard errors of the mean (SEM), calculated by dividing the fluorescence intensity value of the mutant sample by that of the wild-type sample (considered 1.0). *, *P* < 0.05; **, *P* < 0.01 (paired two-tailed Student *t* test). (F) Serial dilution spot analysis to assess the stress susceptibility of the indicated C. glabrata strains in the CAA medium containing Congo red (CR; 2 mg/ml), calcofluor white (CFW; 2 mg/ml), and caffeine (10 mM). The growth of all spot assays was recorded after 1 to 2 days of incubation at 30°C.

Phenotypic analysis of *Cgfig4Δ*, *Cgvac7Δ*, and *Cgvac14Δ* mutants revealed attenuated growth of *Cgvac7Δ* and *Cgvac14Δ* mutants in the presence of the echinocandin antifungal, caspofungin (Fig. 1A). Consistent with our previous study (17), these mutants also displayed increased susceptibility to the azole antifungal, fluconazole (Fig. 1B). Similar to the mutant carrying Tn*7* insertion in the *CgFAB1* gene (17), the *Cgfab1Δ* deletion strain also displayed sensitivity to caspofungin (Fig. 1A). In contrast, growth of the *Cgfig4Δ* mutant largely remained unaffected in azole- and echinocandin-containing medium (Fig. 1A and B).

The cellular response to caspofungin in C. glabrata involves remodelling of the cell wall architecture and upregulation of the chitin synthase gene with a concomitant increase in the cell wall chitin content (10, 32). To investigate whether caspofungin sensitivity of *Cgfab1Δ*, *Cgvac7Δ*, and *Cgvac14Δ* mutants is due to altered cell wall composition, we measured levels of all three components of the cell wall, i.e., β-glucan, mannan, and chitin. Compared to wild-type (wt) cells, we found larger amounts of chitin in the cell walls of *Cgfab1Δ*, *Cgvac7Δ*, and *Cgvac14Δ* mutants (Fig. 1C). Importantly, total β-glucan and mannan levels were not appreciably altered in these mutants (Fig. 1D and E). Consistent with the cell wall defect, *Cgfab1Δ*, *Cgvac7Δ*, and *Cgvac14Δ* mutants also displayed increased sensitivity to cell wall stressors, caffeine (causes generalized cell wall stress), Congo red (binds to β-glucan fibrils), and calcofluor white (impedes chitin assembly) (Fig. 1F). However, these cell wall perturbants had no significant effect on the growth of the *Cgfig4Δ* mutant (Fig. 1F). Furthermore, in agreement with unaltered susceptibility to cell wall stressors and antifungals, the cell wall composition of the *Cgfig4Δ* mutant was found to be similar to that of the wt cell wall (Fig. 1C to E). Ectopic expression of *CgFAB1*, *CgVAC7*, and *CgVAC14* genes in respective mutants restored wild-type-like growth and cell wall composition profiles (Fig. 1A to C and F), indicating that the observed defects in mutants are due to the lack of PI(3,5)P2 complex constituent-encoding genes. Collectively, these data indicate that three main components of the PI(3,5)P2 regulatory complex, CgFab1, CgVac7, and CgVac14, are required for maintenance of the cell wall composition and survival of the cell wall and caspofungin stress in C. glabrata.

### Deletion of *CgVAC7* and *CgVAC14* genes render cells susceptible to surplus metal ion stress.

**FIG 2:**
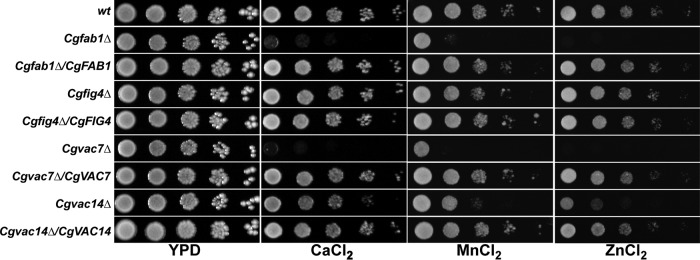
Serial dilution spot analysis to assess the metal ion susceptibility. The indicated overnight-grown C. glabrata strains were 10-fold serially diluted, and 3 μl of each dilution was spotted on medium lacking (YPD) or containing calcium chloride (CaCl_2_; 500 mM), manganese chloride (MnCl_2_; 3 mM), and zinc chloride (ZnCl_2_; 8 mM).

CgFab1 is involved in the regulation of vacuolar functions (17). Since the lack of *CgFAB1*, *CgVAC7*, and *CgVAC14* had a similar effect on cell wall content and stress susceptibility (Fig. 1) and vacuolar morphology (17), we next examined another major phenotype associated with the dysfunctional vacuole, i.e., metal ion storage defect, in *Cgvac7Δ* and *Cgvac14Δ* mutants. We observed a significantly higher sensitivity of *Cgvac7Δ* and *Cgvac14Δ* mutants to excess calcium, manganese, and zinc metal ions (Fig. 2). Further, similar to the cell wall-related phenotypes, the *Cgfig4Δ* mutant displayed wild-type-like growth in the presence of surplus metal ions (Fig. 2). Of note, although enlarged vacuoles, consistent with a previous report (17) were observed in log-phase *Cgfab1Δ*, *Cgvac7Δ*, and *Cgvac14Δ* cells, the *Cgfig4Δ* mutant contained relatively smaller vacuoles (Fig. S1B). Altogether, these data indicate that lack of CgVac7 and CgVac14 perturbs vacuolar size and functions.

### Vacuolar localization of CgFab1 does not require CgVac7, CgVac14, or CgFig4.

**FIG 3:**
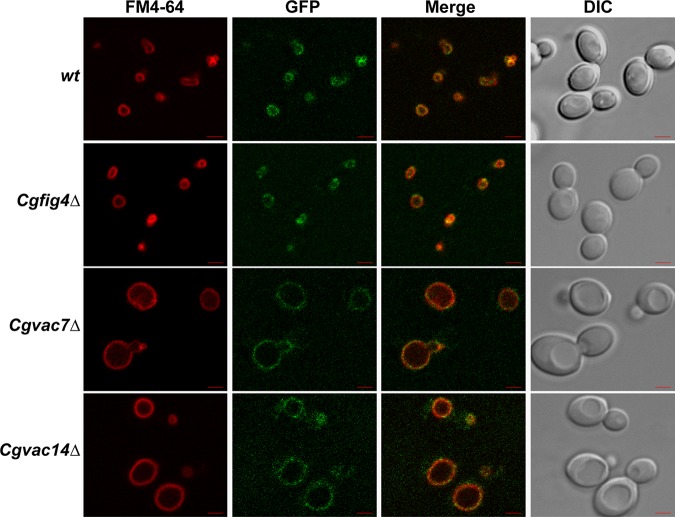
Vacuolar localization of CgFab1 is not impaired in *Cgfig4Δ*, *Cgvac7Δ*, and *Cgvac14Δ* mutants. Representative confocal microscopy images of FM4-64 stained, CAA-grown C. glabrata strains expressing CgFab1-GFP ectopically are shown. GFP, green fluorescent protein; DIC, differential interference contrast. Scale bar, 2.0 μm.

**FIG 4:**
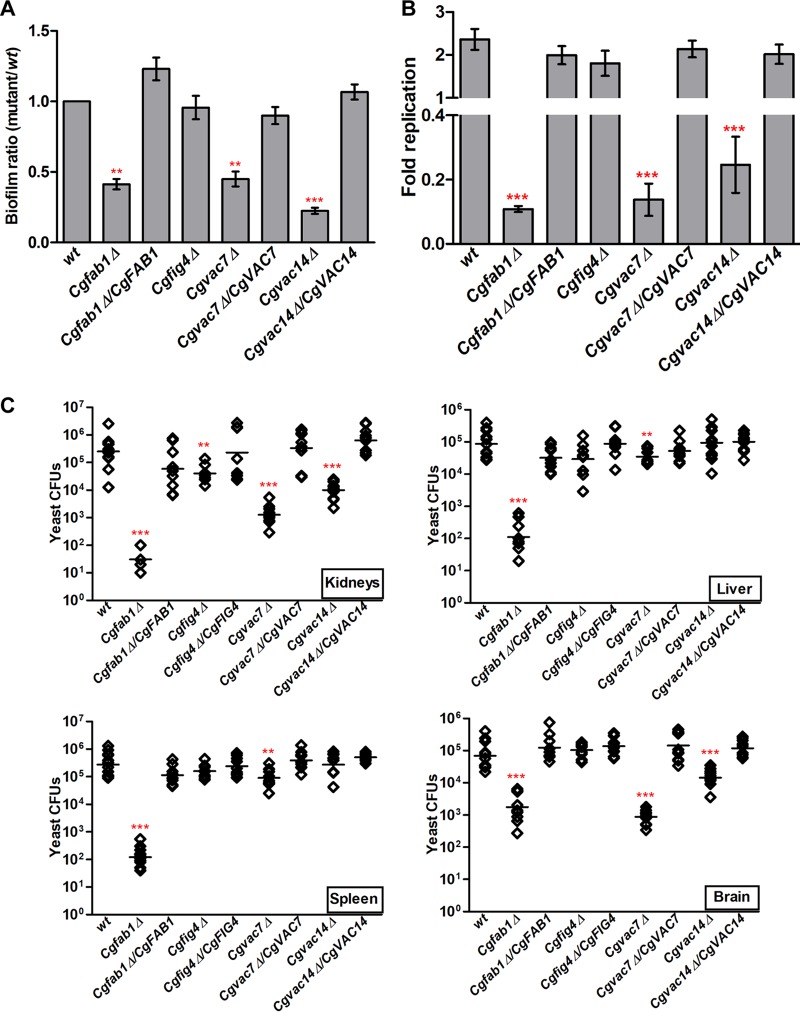
Components of the PI(3,5)P2 regulatory complex are required for virulence. (A) Biofilm formation on polystyrene-coated plates, as measured by crystal violet/ethanol-based staining or destaining. The biofilm ratio was calculated by dividing mutant absorbance units by those of wt cells (set to 1.0; mean ± the SEM; *n* = 3 to 5). **, *P* < 0.01; ***, *P* < 0.001 (paired two-tailed Student *t* test). AU, arbitrary units. (B) CFU-based proliferation analysis of indicated C. glabrata strains in mouse peritoneal macrophages. Infected macrophages were lysed at 2 and 24 h postinfection, and appropriate dilutions of macrophage lysates were plated on YPD medium. The fold replication was calculated by dividing the number of intracellular C. glabrata cells recovered at 24 h by that recovered at 2 h for each strain. Data represent mean ± SEM (*n* = 3 to 4). ***, *P* < 0.001 (unpaired two-tailed Student *t* test). (C) CFU-based survival analysis of the indicated C. glabrata strains in a murine model of systemic candidiasis. After 7 days of intravenous infection with 4 × 10^7^ cells of the indicated C. glabrata strains, the organ fungal load in female BALB/c mice was calculated. Diamonds and bars represent the CFU recovered from target organs of the individual mouse and the CFU geometric mean (*n* = 7 to 13), respectively, for each organ. **, *P* < 0.01; ***, *P* < 0.001 (Mann-Whitney test). Of note, no CFUs were recovered from the kidneys of five *Cgfab1Δ* mutant-infected mice.

Fab1 has previously been shown to be localized to the vacuole in both S. cerevisiae (23) and C. glabrata (17). However, although Fig4 and Vac14 are required for the vacuolar localization of Fab1 in S. cerevisiae (31), their requirement for CgFab1 localization in C. glabrata remains unknown. Therefore, we next stained the vacuolar membranes of wt, *Cgfig4Δ*, *Cgvac7Δ*, and *Cgvac14Δ* cells with the lipophilic dye FM4-64 and checked the localization of CgFab1-green fluorescent protein (GFP) in these strains. We found CgFab1-GFP to be located on the vacuolar membrane in all four strains (Fig. 3), indicating that *CgFIG4*, *CgVAC7*, and *CgVAC14* genes are dispensable for the vacuolar localization of CgFab1 in C. glabrata. These data raise the possibility that despite sharing functional similarity with their S. cerevisiae counterparts, component assembly and/or trafficking of the PI(3,5)P2 regulatory system may follow a different path in C. glabrata.

### CgFab1, CgVac7, and CgVac14 are essential for survival in mice.

An intact cell wall and a functional vacuole are pivotal to the virulence of fungal pathogens (33, 34). Since *Cgfab1Δ*, *Cgvac7Δ*, and *Cgvac14Δ* mutants have defective cell wall and impaired vacuolar functions, we next examined their virulence potential. For this, we first checked their ability to form biofilms *in vitro* and found highly diminished biofilms formed by *Cgfab1Δ*, *Cgvac7Δ*, and *Cgvac14Δ* mutants on polystyrene, compared to wt biofilms (Fig. 4A). C. glabrata is known to proliferate in primary macrophages *in vitro* (35). Hence, we next determined the intracellular replication profiles of *Cgfab1Δ*, *Cgvac7Δ*, *Cgvac14Δ*, and *Cgfig4Δ* mutants in mouse macrophages. We observed that while wt and *Cgfig4Δ* cells multiplied in mouse primary peritoneal macrophages, only 10 to 20% cells of *Cgfab1Δ*, *Cgvac7Δ*, and *Cgvac14Δ* mutants survived after 24 h coculture with primary macrophages (Fig. 4B). Lastly, we checked the ability of *Cgfab1Δ*, *Cgvac7Δ*, *Cgvac14Δ*, and *Cgfig4Δ* mutants to survive in the murine model of systemic candidiasis. Similar to the *in vitro* results, ca. 0.4 to 4 log units fewer CFUs were recovered for *Cgfab1Δ* and *Cgvac7Δ* mutants, compared to the wt strain, from the target organs, i.e., the kidneys, livers, spleens, and brains of infected mice (Fig. 4C). Mice infected with the *Cgfig4Δ* mutant displayed 13-fold-reduced renal fungal burden compared to wt-infected mice (Fig. 4C). In contrast, 5- and 25- fold fewer CFUs were recovered from the brains and kidneys, respectively, of the *Cgvac14Δ*-infected mice compared to the *Cgfig4Δ* mutant (Fig. 4C). Importantly, all the defects listed above for the *Cgfab1Δ*, *Cgfig4Δ*, *Cgvac7Δ*, and *Cgvac14Δ* mutants were rescued upon ectopic expression of the respective genes (Fig. 4A to C). These data demonstrate that CgFab1, CgVac7, and CgVac14 are pivotal to the pathogenesis of C. glabrata.

### *CgFIG4* deletion in *Cgfab1Δ* and *Cgvac7Δ* mutants rescues stress sensitivity.

**FIG 5:**
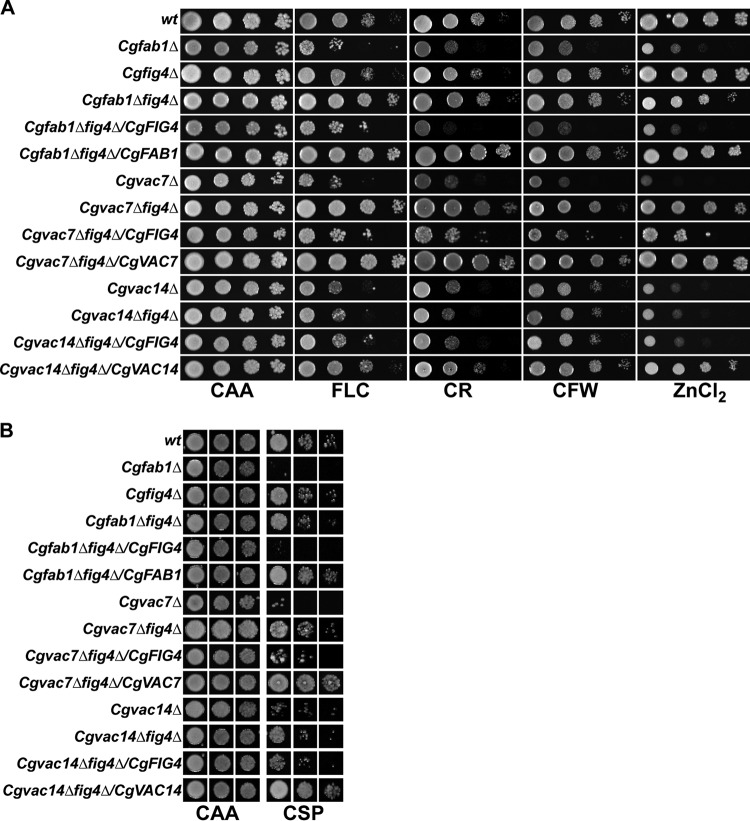
*CgFIG4* loss rescues stress susceptibility of *Cgfab1Δ* and *Cgvac7Δ* mutants. (A) Serial dilution spot analysis to assess the stress susceptibility of indicated C. glabrata strains in CAA medium containing fluconazole (FLC; 16 μg/ml), Congo red (CR; 2 mg/ml), calcofluor white (CFW; 2 mg/ml), and zinc chloride (ZnCl_2_; 8 mM). (B) Liquid growth assay-based analysis of indicated C. glabrata strains in the medium lacking (CAA) or containing caspofungin (CSP; 75 ng/ml).

After demonstrating their centrality to antifungal tolerance, cell wall metabolism, and virulence, we next sought to decipher the relationship between different components of the PI(3,5)P2 regulatory complex, CgFab1, CgVac7, CgVac14, and CgFig4. For this, we took two genetic approaches. First, we deleted the *CgFIG4* gene in all three mutants, *Cgfab1Δ*, *Cgvac7Δ*, and *Cgvac14Δ*, since *Fig4* in S. cerevisiae is known to regulate PI(3,5)P2 levels (29). Our rationale was that if the phenotypes of the *Cgfab1Δ*, *Cgvac7Δ*, and *Cgvac14Δ* mutants are due to low levels of PI(3,5)P2, the absence of CgFig4 may result in stabilization and relatively higher PI(3,5)P2 levels, thereby improving the mutants’ stress tolerance. Comprehensive phenotypic characterization of double mutants revealed that *Cgfab1Δfig4Δ* and *Cgvac7Δfig4Δ* mutants showed wild-type-like growth in media supplemented with fluconazole, Congo red, calcofluor white, or zinc chloride (Fig. 5A) and caspofungin (Fig. 5B). Importantly, *CgFIG4* expression in double deletion mutants brought back the original stress susceptibility of the single mutant, thereby unequivocally linking the stress resistance phenotype with the absence of CgFig4 (Fig. 5). Expectedly, expression of *CgFAB1* and *CgVAC7* in *Cgfab1Δfig4Δ* and *Cgvac7Δfig4Δ* mutants, respectively, resulted in wild-type-like growth in media containing antifungal drugs, cell wall stressors, and zinc chloride (Fig. 5).

**FIG 6:**
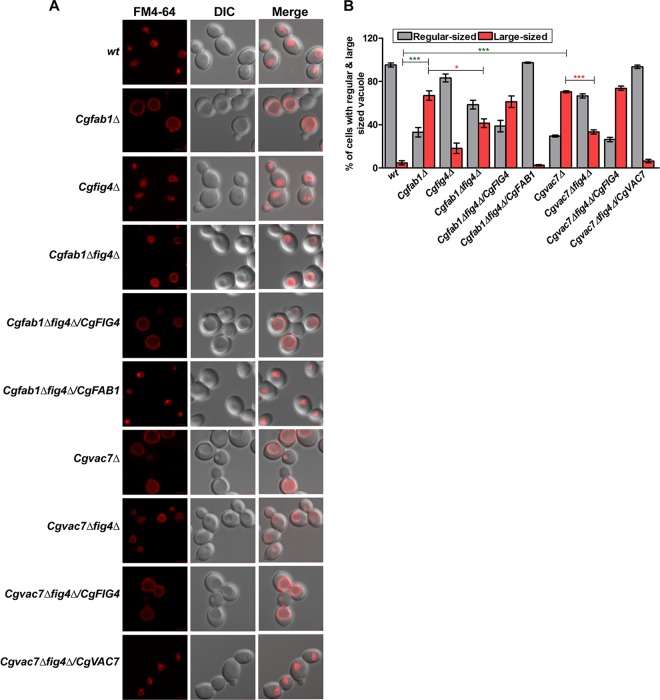
*CgFIG4* deletion restores the large-vacuole phenotype of *Cgfab1Δ* and *Cgvac7Δ* mutants. (A) Representative confocal microscopy images of FM4-64 stained, YPD-grown log-phase cultures of indicated C. glabrata strains are shown. DIC, differential interference contrast. Scale bar, 2.0 μm. (B) Quantification of cell population carrying a large vacuole. A minimum of 150 cells displaying FM4-64 staining of the vacuole for each strain were counted, and data are presented as the percentage of cells containing regular-sized and large vacuoles. *, *P* < 0.05; **, *P* < 0.01; ***, *P* < 0.001 (unpaired, two-tailed, Student *t* test).

Next, we checked the effect of *CgFIG4* deletion on the vacuole size and found a partial reversal of the large vacuole phenotype of *Cgfab1Δ* and *Cgvac7Δ* mutants (Fig. 6). In contrast, the *Cgvac14Δfig4Δ* mutant contained a large vacuole (Fig. S2) and exhibited sensitivity, similar to the *Cgvac14Δ* mutant, to fluconazole, Congo red, calcofluor white, or zinc chloride (Fig. 5A) and caspofungin (Fig. 5B). Altogether, these data suggest that CgFig4 deletion probably stabilizes and maintains PI(3,5)P2 levels above a certain threshold value, resulting in the rescue of defects associated with the absence of the PI(3,5)P2-generating factors CgFab1 and CgVac7. However, nonrestoration of the increased stress susceptibility of the *Cgvac14Δ* mutant, upon CgFig4 loss, indicates that either *Cgvac14Δ* phenotypes are not solely due to small amounts of PI(3,5)P2 or that CgVac14 is required for CgFig4 activity.

### *CgFAB1* and *CgVAC7* overexpression restores the stress sensitivity of the *Cgvac14Δ* mutant.

**FIG 7:**
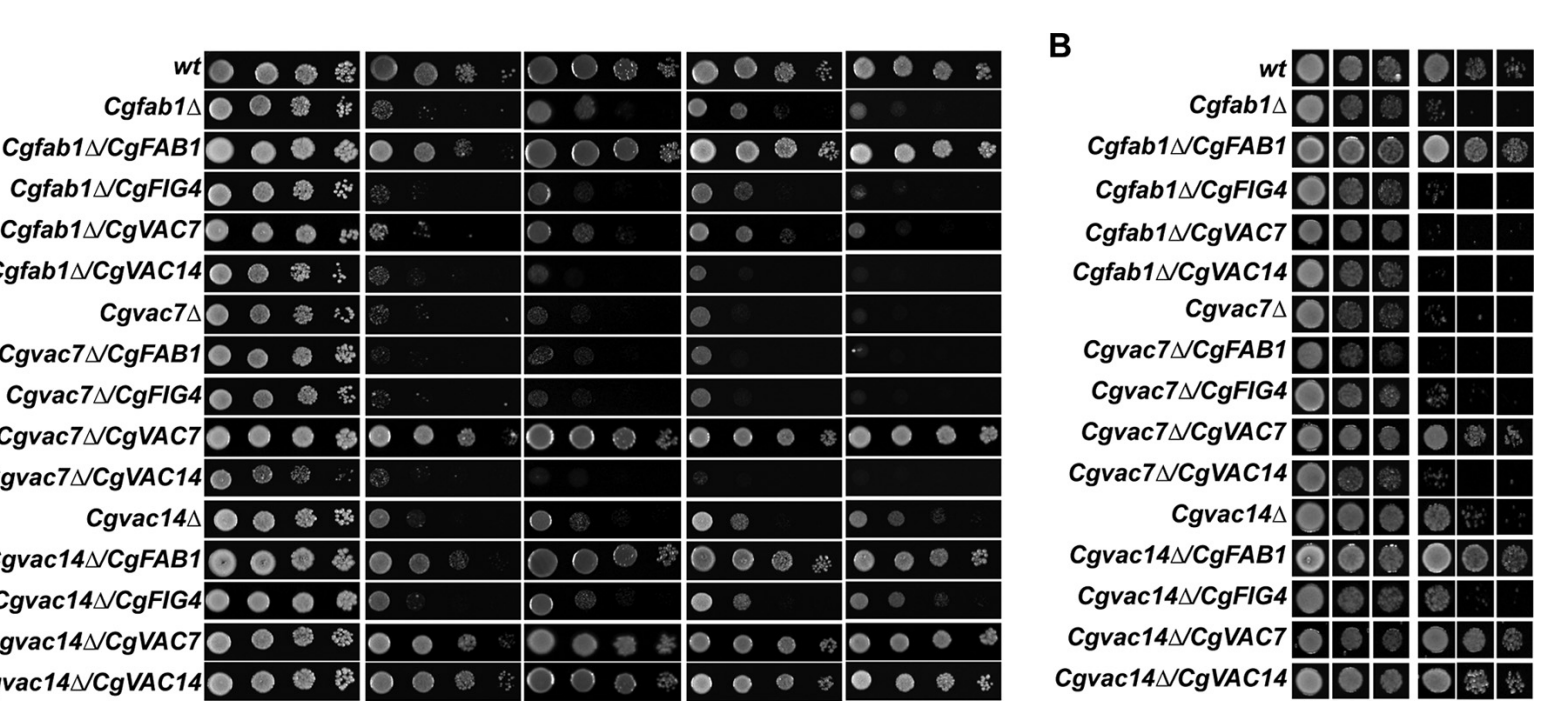
*CgFAB1* and *CgVAC7* overexpression rescues stress susceptibility of the *Cgvac14Δ* mutant. (A) Serial dilution spot analysis to assess the susceptibility of indicated C. glabrata strains toward fluconazole (FLC; 16 μg/ml), Congo red (CR; 2 mg/ml), calcofluor white (CFW; 2 mg/ml), and zinc chloride (ZnCl_2_; 8 mM). (B) Liquid growth assay-based analysis of indicated C. glabrata strains in the medium lacking (CAA) or containing caspofungin (CSP; 75 ng/ml).

The second genetic approach that we undertook involved the overexpression of all four genes, *CgFAB1*, *CgFIG4*, *CgVAC7*, and *CgVAC14*, individually in wt, *Cgfab1Δ*, *Cgfig4Δ*, *Cgvac7Δ*, and *Cgvac14Δ* mutants, with the rationale that overexpression of PI(3,5)P2 synthesis complex proteins (CgFab1, CgVac7, and CgVac14) may lead to increased PI(3,5)P2 levels. Phenotypic analysis revealed that overexpression of *CgFAB1*, *CgFIG4*, *CgVAC7*, and *CgVAC14* genes in the wt had no significant effect on tolerance to fluconazole and caspofungin (Fig. S2A and B). Moreover, the growth of wt cells expressing these genes also remained largely unaffected in the presence of cell wall stressors and excess zinc chloride (Fig. S3A). Similarly, *CgFAB1*, *CgVAC7*, and *CgVAC14* overexpression in the *Cgfig4Δ* mutant had no discernible phenotype (Fig. S3A and B). Overexpression of *CgFIG4* also had no effect on stress susceptibility of *Cgfab1Δ*, *Cgvac7Δ*, and *Cgvac14Δ* mutants (Fig. 7). Strikingly, overexpression of *CgFAB1* and *CgVAC7* rescued the azole, caspofungin, Congo red, calcofluor white, and surplus zinc ion sensitivity of the *Cgvac14Δ* mutant (Fig. 7). However, the converse effect was not observed, as *CgVAC14* overexpression could not restore susceptibility of *Cgfab1Δ* and *Cgvac7Δ* mutants to various stresses (Fig. 7). Similarly, neither *CgFAB1* nor *CgVAC7* overexpression could rescue growth defect of the *Cgvac7Δ* and *Cgfab1Δ* mutant, respectively, in antifungal-, cell wall stressor-, and zinc-containing medium (Fig. 7). Altogether, four key findings emerge from these results. First, enhanced stress susceptibility of the *Cgvac14Δ* mutant is likely to be due to low PI(3,5)P2 levels, thereby implicating CgVac14 in regulation of CgFab1 activity. Second, CgVac7 is most likely a positive regulator of CgFab1 kinase activity and/or PI(3,5)P2 level maintenance in C. glabrata. Third, CgFab1 requires CgVac7 for its activity. Finally, PI(3,5)P2 levels are probably very tightly controlled in C. glabrata, since none of the overexpressed PI(3,5)P2 regulatory complex component could alter the cellular PI(3,5)P2 levels significantly enough to impact antifungal tolerance. It is possible that CgFab1 is subjected to feedback inhibition control.

**FIG 8:**
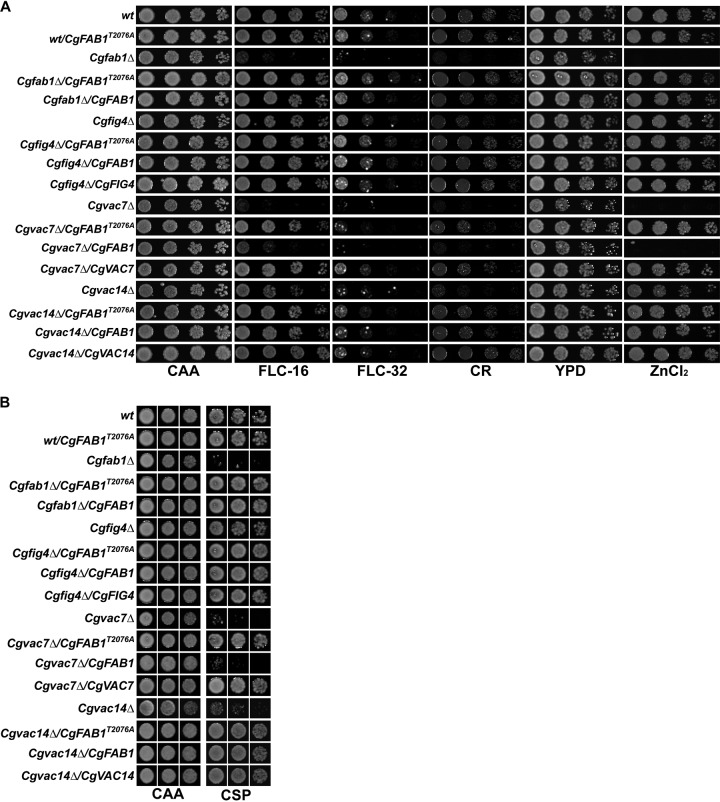
The constitutively active CgFab1 enzyme restores the stress susceptibility of *Cgvac7Δ* and *Cgvac14Δ* mutants. (A) Serial dilution spot analysis to assess the susceptibility of indicated C. glabrata strains toward fluconazole (FLC; 16 and 32 μg/ml), Congo red (CR, 2 mg/ml), and zinc chloride (ZnCl_2_; 8 mM). Of note, sensitivity to fluconazole/Congo red and zinc chloride was checked in CAA and YPD media, respectively. (B) Liquid growth assay-based analysis of the indicated C. glabrata strains in medium lacking (CAA) or containing caspofungin (CSP; 75 ng/ml).

Next, we sought to verify that PI(3,5)P2 levels indeed regulate stress tolerance in C. glabrata. Since we were unable to measure PI(3,5)P2 levels biochemically due to technical constraints, we decided to address the issue genetically. Fab1 in S. cerevisiae is a well-studied enzyme, and a single amino acid mutation is known to bypass the activity regulatory mechanisms, resulting in a constitutively active enzyme (23, 27). Hence, we aligned the amino acid sequence of CgFab1 with ScFab1 and identified the highly conserved threonine residue that renders ScFab1 constitutively active (Fig. S4A). We constructed the variant CgFab1 protein by substituting threonine at 2076 position with alanine. In contrast to our expectations, CgFab1^T2076A^ overexpression did not lead to resistance toward azole and caspofungin in wt cells (Fig. 8), indicating a very tight regulation of PI(3,5)P2 levels in C. glabrata. However, overexpression of this CgFab1 enzyme rescued the fluconazole, Congo red, zinc chloride, and caspofungin sensitivity (Fig. 8), as well as the large vacuole phenotype of both *Cgvac7Δ* and *Cgvac14Δ* mutants (Fig. S4B), suggesting that the dysfunctional vacuole and increased susceptibility to cell wall and antifungal stress of *Cgvac7Δ* and *Cgvac14Δ* mutants is *de facto* due to lower PI(3,5)P2 levels in these mutants. These data are also in agreement with the earlier finding that CgVac7 is required for activation of the lipid kinase activity of CgFab1, since the overexpression of the wild-type allele of *CgFAB1* could neither restore the increased stress susceptibility nor the malfunctioning vacuole (Fig. 7).

### The PI(3,5)P2 synthesis system uniquely regulates caspofungin tolerance.

Since the loss of any one of the components of the PI(3,5)P2 synthesis complex led to increased sensitivity to caspofungin, we next examined whether subunits of the phosphoinositide 3-kinase (PI3K) complex are also required for caspofungin tolerance. The regulatory and catalytic components of the PI3K complex are represented by CgVps15 and CgVps34, respectively (35). While CgVps34 has been shown to be a lipid kinase, which converts phosphatidylinositol to phosphatidylinositol 3-phosphate, CgVps15 is predicted to be a serine/threonine protein kinase (35). Both *Cgvps15Δ* and *Cgvps34Δ* mutants possess large vacuoles and are essential for the virulence in C. glabrata (35). We checked the caspofungin sensitivity of *Cgvps15Δ* and *Cgvps34Δ* mutants by using a liquid growth assay and found them to grow well in the caspofungin-containing medium (Fig. S5), indicating that PI3P signaling does not modulate the cellular response to caspofungin. These data underscore a unique role for PI(3,5)P2 lipid in caspofungin tolerance in C. glabrata.

## DISCUSSION

The pathogenic yeast C. glabrata possesses orthologs of all components of the S. cerevisiae PI(3,5)P2 regulatory complex: Fab1, Vac7, Vac14, and Atg18. C. glabrata Fab1, Vac7, Vac14, and Atg18 are 2,104-, 1,076-, 868-, and 505-amino-acid proteins, respectively (Fig. S1A) and show 35 to 70% identity with their S. cerevisiae counterparts. Importantly, the domains involved in the regulation of PI(3,5)P2 levels are conserved between proteins of these two yeasts (Fig. S1A). In the present study, we show that components of the PI(3,5)P2 signaling pathway are required for cell wall and vacuole homeostasis and antifungal tolerance in C. glabrata. Despite PI(3,5)P2 being a low-abundance phosphoinositide (24, 26), we demonstrate for the first time that the PI(3,5)P2-generating kinase, CgFab1, is essential for the virulence of C. glabrata. Similarly, CgFig4, CgVac7, and CgVac14 proteins, which regulate PI(3,5)P2 levels, were also required for C. glabrata pathogenesis.

Besides the dysfunctional large vacuole, lack of *CgFAB1*, *CgVAC7*, and *CgVAC14* also resulted in higher cell wall chitin content that may stem from impaired trafficking of the cell wall biogenesis proteins, since PI(3,5)P2 levels are known to regulate protein trafficking events in both lower and higher eukaryotes (24–26). Although these data raise the possibility that both the dysfunctional vacuole and the altered/fragile cell wall may contribute to the increased caspofungin sensitivity of *Cgfab1Δ*, *Cgvac7Δ*, and *Cgvac14Δ* mutants, the wild-type-like caspofungin susceptibility of *Cgvps15Δ* and *Cgvps34Δ* mutants, despite having a malfunctioning vacuole (35), renders the first prospect unlikely. Elucidation of the molecular link between high cell wall chitin and caspofungin sensitivity of *Cgfab1Δ*, *Cgvac7Δ*, and *Cgvac14Δ* mutants will help elucidate the mechanism underlying the role of PI(3,5)P2 in echinocandin tolerance.

Further, our data suggest that the mechanisms of assembly and trafficking of PI(3,5)P2 complex in C. glabrata and S. cerevisiae are disparate. In contrast to C. glabrata, localization of Fab1 to the vacuole in S. cerevisiae is dependent upon both Vac14 and Fig4 (31). Similarly, functions of the phosphoinositide-binding protein Atg18, which acts as a negative regulator of Fab1 in S. cerevisiae (36), appear to be not conserved between S. cerevisiae and C. glabrata, since *CgATG18* deletion did not significantly alter the vacuole size in C. glabrata (Fig. S6A). The growth of the *Cgatg18Δ* mutant was also found to be not attenuated in the presence of surplus metal ions, cell wall stressors, and antifungal drugs (Fig. S6B and C). Therefore, investigations focusing on physical interaction between different components of the C. glabrata PI(3,5)P2 complex are warranted in order to gain mechanistic insights into the complex biogenesis.

**FIG 9:**
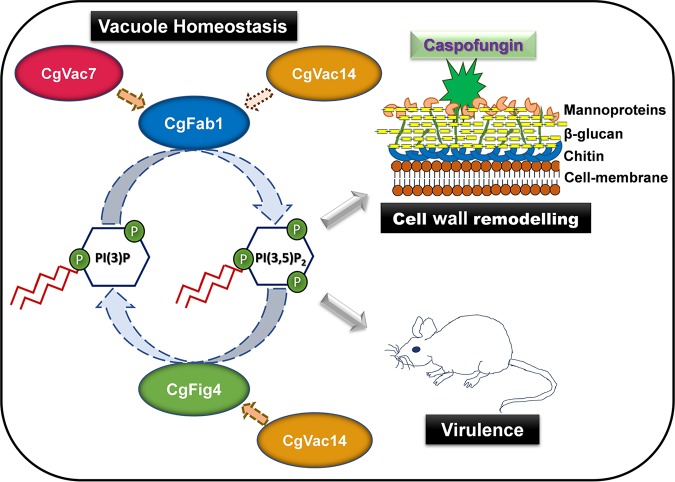
Schematic summary of key results of this study. Cellular PI(3,5)P2 levels in C. glabrata regulate vacuole homeostasis, caspofungin tolerance, and virulence in mice. CgFab1 is a PI(3,5)P2 kinase that converts PI3P to PI(3,5)P2. CgVac7 and CgVac14 positively regulate the activity of CgFab1, as indicated by dotted arrows. CgFig4 is PI(3,5)P2 phosphatase that converts PI(3,5)P2 to PI3P, and its activity is modulated by CgVac14. Altogether, CgFab1, CgVac7, CgVac14, and CgFig4 maintain PI(3,5)P2 levels in C. glabrata.

Altogether, our data uncover the functions and activity regulatory mechanisms of the PI(3,5)P2 complex (Fig. 9) for the first time in C. glabrata. Our genetic and phenotypic analyses provide evidence for the following four major conclusions: (i) the dysfunctional vacuole and increased antifungal and cell wall stress susceptibility of *Cgvac7Δ* and *Cgvac14Δ* mutants is due to low PI(3,5)P2 levels; (ii) the PI(3,5)P2 levels are regulated positively and negatively by CgVac7 and CgFig4, respectively; (iii) despite the dispensability for CgFab1 localization, CgVac7 is essential for activation of the PI3P 5-kinase activity of CgFab1; and (iv) CgVac14 may be pivotal to regulate the phosphatidylinositol-3,5-bisphosphate 5-phosphatase [PI(3,5)P2 5-phosphatase] activity of CgFig4 (Fig. 9). Overall, our results underscore the complex and distinct genetic relationships among PI(3,5)P2 kinase, PI(3,5)P2 phosphatase, and positive regulators of PI(3,5)P2 kinase and phosphatase and the effect of this relationship on tolerance to azole and echinocandin antifungals.

In light of an essential role for PI(3,5)P2 signaling in antifungal tolerance and virulence of C. glabrata, we delved into the possibility of fungal Vac7 as a novel antifungal target. Consistent with an earlier report (26), our BLAST analysis identified no homologs of CgVac7 in humans and mice. Further, multiple amino acid sequence alignment of Vac7 of seven fungal species (Aspergillus fumigatus, C. albicans, C. glabrata, C. parapsilosis, C. tropicalis, Histoplasma capsulatum, and S. cerevisiae) revealed three weakly conserved regions (Fig. S7). Of these, two regions fall in the vacuolar segregation domains of CgVac7 (Fig. S1A and S7). Although our preliminary analysis favors Vac7 as an antifungal target, detailed structural modeling and inhibitor studies are needed to test this possibility completely.

In summary, in addition to characterizing components of the PI(3,5)P2 regulatory complex in C. glabrata for the first time, our study opens up a new avenue, using the absence of Vac7 homologs in higher eukaryotes, for antifungal therapy.

## MATERIALS AND METHODS

### Strains and media.

C. glabrata strains, derivatives of the C. glabrata BG2 strain, were routinely grown in the rich yeast extract-peptone-dextrose (YPD) medium or minimal yeast nitrogen base medium containing Casamino Acid (CAA) at 30°C. The Escherichia coli DH5α strain was used for gene cloning and grown in the LB medium at 37°C. Logarithmic-phase yeast cells were obtained by incubating the overnight grown cultures in the fresh YPD/CAA medium for about 4 h.

### Animal experiments.

Mice infection experiments were performed at the Centre for DNA Fingerprinting and Diagnostics (CDFD) Animal House Facility and VIMTA Labs Limited, Hyderabad, India, in accordance with guidelines of the Committee for the Purpose of Control and Supervision of Experiments on Animals, Government of India. The protocols were approved by the Institutional Animal Ethics Committee.

### Isolation of primary (peritoneal) macrophages.

Six- to eight-week-old BALB/c mice were used to isolate primary macrophages, as described previously (35). 1.5 ml of 4% (wt/vol) thioglycolate broth was injected into the peritoneal cavity of mice. After 5 to 7 days, mice were euthanized by CO_2_ inhalation, and peritoneal macrophages were collected by peritoneal lavage with 10 ml of Dulbecco modified Eagle medium (DMEM), followed by centrifugation. After two washes, the cells were suspended in DMEM containing heat-inactivated fetal bovine serum (10%), glutamine (2 mM), penicillin (100 U/ml), and streptomycin (100 μg/ml) and seeded in a 24-well culture plate at a density 1 × 10^6^ per well. After 24 h of incubation at 37°C and 5% CO_2_, the macrophages were infected with C. glabrata cells at a multiplicity of infection of 0.1, as described previously (35).

### *C. glabrata* gene disruption and cloning.

The *CgFIG4* (*CAGL0D02464g*, 2.6 kb) gene at the genomic loci was replaced, using a homologous-recombination-based strategy, with the *NAT1* cassette, as described previously (14). For the creation of double-deletion strains, the parental single-deletion strain (*Cgfab1Δ*, *Cgvac7Δ*, or *Cgvac14Δ*) was transformed with the pRK70 plasmid expressing the FLP flippase, and transformants were selected for uracil prototrophy in the CAA medium. The flippase enzyme facilitates the recombination at FRT sites flanking the *nat1* gene resulting in the *NAT1* cassette excision. The nourseothricin-sensitive transformants were purified and cured of the plasmid pRK70 after four to five passages in nonselective YPD medium. The plasmid curing was verified by growth on 5-fluroorotic acid, and cells were transformed with the linearized fragment, PCR amplified from the genomic DNA of *Cgfig4Δ* deletion strain, carrying *CgFIG4* 5′-untranslated region (5′-UTR)-*NAT1* cassette-*CgFIG4* 3′-UTR sequence. Nourseothricin-resistant colonies were purified and checked for gene disruption by PCR.

For complemented strain generation, *CgFIG4*, *CgVAC7* (*CAGL0G01342g*, 3.23 kb), and *CgVAC14* (*CAGL0H06501g*, 2.6 kb) were PCR amplified from the wild-type genomic DNA using the high-fidelity Phusion *Taq* polymerase and cloned downstream of the *PGK1* promoter at SpeI-BamHI, EcoRI-XhoI, and SpeI-EcoRI restriction enzyme sites, respectively, in the pGRB2.2 (pRK74) plasmid. The recombinant plasmids were expressed in the corresponding deletion mutants for complementation analysis.

The hyperactive allele of CgFab1 was created by replacing the conserved threonine amino acid at position 2076 with the alanine using the site-directed mutagenesis as described previously (17). The mutation was confirmed by sequencing. For overexpression studies, PCR amplified *CgFAB1*, *CgFIG4*, *CgVAC7*, and *CgVAC14* genes were cloned downstream of the high-strength *PDC1* promoter at the XmaI-SalI, SpeI-XmaI, XmaI-EcoRI, and SpeI-EcoRI sites, respectively, in pCU-PDC1 plasmids and expressed in C. glabrata strains. All strains/plasmids and primers used in this study are listed in Tables S1 and S2, respectively.

### Serial dilution spot assay.

The serial dilution spotting assay was performed in solid medium to assess the susceptibility of C. glabrata strains to fluconazole, metal ions, and cell wall and cell membrane stress. Briefly, overnight-grown cultures were normalized to an optical density at 600 nm (OD_600_) of 1.0- and 10-fold serially diluted in phosphate-buffered saline (PBS). Then, 3 μl of each dilution was spotted onto the appropriate medium, and growth was recorded after 1 to 2 days of incubation at 30°C. For caspofungin sensitivity analysis, the liquid growth susceptibility assay was performed in a 96-well plate containing 100 μl of CAA medium with or without the drug. Overnight-grown C. glabrata cultures were inoculated in this medium at an OD_600_ of 0.2 and grown for 24 h at 30°C with constant shaking. The cultures were diluted 25-, 125-, and 625- fold, and 3 μl of each dilution was spotted onto CAA agar medium.

### Chitin, β-glucan, and mannan estimation.

Log-phase-grown C. glabrata cultures were harvested, washed with PBS, and suspended in 1 ml of PBS to obtain a final OD_600_ of 2.0. To this suspension, calcofluor white (2.5 μg/ml), aniline blue (12.5 μg/ml), and fluorescein isothiocyanate-labeled concanavalin A (1 μg/ml) were added for chitin, β-glucan, and mannan estimation, respectively. Cells were incubated in the dark for 15 min at room temperature and washed twice with PBS. About 50,000 fluorescently labeled and unlabeled cells were analyzed using the flow cytometry (BD FACSAria III). The mean fluorescence intensity value, calculated using FlowJo software, for unstained control cells was subtracted from that for stained cells, and data were plotted as the mean intensity ratio, which reflected the mutant/wild-type fluorescence intensity values.

### Microscopy.

Vacuolar morphology in C. glabrata cells was visualized using the lipophilic dye FM4-64, which is known to be endocytosed by the plasma membrane. Log-phase-grown cells (1 ml) were harvested, washed, and suspended in YPD medium containing FM4-64 (16 μM). After 30 min of incubation at 30°C in the dark, the cells were washed twice and incubated in the fresh YPD medium for 90 min. After two washes with PBS, the cells were suspended in CAA medium and visualized under a confocal microscope (Zeiss LSM 700; 63×/1.44 NA objective; Leica TCS SP8; 63×/1.52NA objective). Representative maximum-intensity projection of Z-stack fluorescence confocal images were prepared by fluorescent image deconvolution, followed by projection of a subset (5 to 10 images) of the entire Z-stack into a single image. Z-stack images were acquired throughout the cell at 0.25 and 0.5 μm intervals for Zeiss and Leica confocal microscope, respectively. CgFab1 localization was studied by fluorescent imaging of log-phase cells expressing GFP-tagged CgFab1 from the pGRB2.3 plasmid.

### Biofilm assay.

The ability of YPD-grown log-phase C. glabrata cells to form biofilms on polystyrene was checked by growing cells to an OD_600_ of 0.5 in a 24-well plate at 37°C. After 90 min of incubation, the cells were washed twice with PBS, suspended in RPMI 1640 medium containing 10% fetal bovine serum, and incubated for another 48 h with a medium change at 24 h. PBS washes were applied to remove unbound cells, and adherent yeast cells were stained with a 0.4% (wt/vol) crystal violet solution for 45 min. C. glabrata biofilms were destained with 95% ethanol, and the destaining solution absorbance was recorded at 595 nm. The absorbance values of blank wells (without yeast) were subtracted from those of C. glabrata-containing wells. The biofilm ratio represents the mutant/wild-type absorbance units.

### Mouse infection assay.

YPD medium-grown C. glabrata cells (4 × 10^7^; 100 μl of PBS cell suspension) were injected into tail vein of 6- to 8-week-old female BALB/c mice. At day 7 postinfection, mice were sacrificed, and four organs—kidney, liver, brain, and spleen—were collected and homogenized in PBS. Appropriate homogenate dilutions were plated on YPD medium supplemented with penicillin and streptomycin to determine the fungal load in mouse organs.

### Statistical analysis.

Statistical significance was determined using GraphPad Prism software. A two-tailed Student *t* test and the nonparametric Mann-Whitney test were used for intergroup comparisons and mouse organ fungal burden analysis, respectively.

## Supplementary Material

Supplemental file 1

## References

[1] Alexander BD, Johnson MD, Pfeiffer CD, Jimenez-Ortigosa C, Catania J, Booker R, Castanheira M, Messer SA, Perlin DS, Pfaller MA 2013 Increasing echinocandin resistance in *Candida glabrata*: clinical failure correlates with presence of FKS mutations and elevated minimum inhibitory concentrations. Clin Infect Dis 56:1724–1732. doi:10.1093/cid/cit136. 23487382 PMC3658363

[2] Pfaller MA, Messer SA, Moet GJ, Jones RN, Castanheira M 2011 *Candida* bloodstream infections: comparison of species distribution and resistance to echinocandin and azole antifungal agents in intensive care unit (ICU) and non-ICU settings in the SENTRY Antimicrobial Surveillance Program (2008-2009). Int J Antimicrob Agents 38:65–69. doi:10.1016/j.ijantimicag.2011.02.016. 21514797

[3] Pfaller MA, Diekema DJ, Turnidge JD, Castanheira M, Jones RN 2019 Twenty years of the SENTRY Antifungal Surveillance Program: results for *Candida* species from 1997 to 2016. Open Forum Infect Dis 6:S79–S94. doi:10.1093/ofid/ofy358. 30895218 PMC6419901

[4] Chakrabarti A, Sood P, Rudramurthy SM, Chen S, Kaur H, Capoor M, Chhina D, Rao R, Eshwara VK, Xess I, Kindo AJ, Umabala P, Savio J, Patel A, Ray U, Mohan S, Iyer R, Chander J, Arora A, Sardana R, Roy I, Appalaraju B, Sharma A, Shetty A, Khanna N, Marak R, Biswas S, Das S, Harish BN, Joshi S, Mendiratta D 2015 Incidence, characteristics, and outcome of ICU-acquired candidemia in India. Intensive Care Med 41:285–295. doi:10.1007/s00134-014-3603-2. 25510301

[5] Pfaller MA, Diekema DJ, Gibbs DL, Newell VA, Barton R, Bijie H, Bille J, Chang S-C, da Luz Martins M, Duse A, Dzierzanowska D, Ellis D, Finquelievich J, Gould I, Gur D, Hoosen A, Lee K, Mallatova N, Mallie M, Peng NK, Petrikos G, Santiago A, Trupl J, VanDen Abeele AM, Wadula J, Zaidi M 2010 Geographic variation in the frequency of isolation and fluconazole and voriconazole susceptibilities of *Candida glabrata*: an assessment from the ARTEMIS DISK Global Antifungal Surveillance Program. Diagn Microbiol Infect Dis 67:162–171. doi:10.1016/j.diagmicrobio.2010.01.002. 20338711

[6] Akins RA 2005 An update on antifungal targets and mechanisms of resistance in *Candida albicans*. Med Mycol 43:285–318. doi:10.1080/13693780500138971. 16110776

[7] Vale-Silva LA, Sanglard D 2015 Tipping the balance both ways: drug resistance and virulence in *Candida glabrata*. FEMS Yeast Res 15:fov025. doi:10.1093/femsyr/fov025. 25979690

[8] Hachem R, Hanna H, Kontoyiannis D, Jiang Y, Raad I 2008 The changing epidemiology of invasive candidiasis. Cancer 112:2493–2499. doi:10.1002/cncr.23466. 18412153

[9] Dellière S, Healey K, Gits-Muselli M, Carrara B, Barbaro A, Guigue N, Lecefel C, Touratier S, Desnos-Ollivier M, Perlin DS, Bretagne S, Alanio A 2016 Fluconazole and echinocandin resistance of *Candida glabrata* correlates better with antifungal drug exposure rather than with MSH2 mutator genotype in a French cohort of patients harboring low rates of resistance. Front Microbiol 7:2038. doi:10.3389/fmicb.2016.02038. 28066361 PMC5179511

[10] Perlin DS 2015 Echinocandin resistance in *Candida*. Clin Infect Dis 61(:Suppl 6):S612–S617. doi:10.1093/cid/civ791. 26567278 PMC4643482

[11] Castanheira M, Messer SA, Rhomberg PR, Pfaller MA 2016 Antifungal susceptibility patterns of a global collection of fungal isolates: results of the SENTRY Antifungal Surveillance Program (2013). Diagn Microbiol Infect Dis 85:200–204. doi:10.1016/j.diagmicrobio.2016.02.009. 27061369

[12] Singh-Babak SD, Babak T, Diezmann S, Hill JA, Xie JL, Chen YL, Poutanen SM, Rennie RP, Heitman J, Cowen LE 2012 Global analysis of the evolution and mechanism of echinocandin resistance in *Candida glabrata*. PLoS Pathog 8:e1002718. doi:10.1371/journal.ppat.1002718. 22615574 PMC3355103

[13] Kaur R, Castano I, Cormack BP 2004 Functional genomic analysis of fluconazole susceptibility in the pathogenic yeast *Candida glabrata*: roles of calcium signaling and mitochondria. Antimicrob Agents Chemother 48:1600–1613. doi:10.1128/aac.48.5.1600-1613.2004. 15105111 PMC400560

[14] Borah S, Shivarathri R, Kaur R 2011 The Rho1 GTPase-activating protein CgBem2 is required for survival of azole stress in *Candida glabrata*. J Biol Chem 286:34311–34324. doi:10.1074/jbc.M111.264671. 21832071 PMC3190821

[15] Schwarzmüller T, Ma B, Hiller E, Istel F, Tscherner M, Brunke S, Ames L, Firon A, Green B, Cabral V, Marcet-Houben M, Jacobsen ID, Quintin J, Seider K, Frohner I, Glaser W, Jungwirth H, Bachellier-Bassi S, Chauvel M, Zeidler U, Ferrandon D, Gabaldón T, Hube B, d’Enfert C, Rupp S, Cormack B, Haynes K, Kuchler K 2014 Systematic phenotyping of a large-scale *Candida glabrata* deletion collection reveals novel antifungal tolerance genes. PLoS Pathog 10:e1004211. doi:10.1371/journal.ppat.1004211. 24945925 PMC4063973

[16] Rosenwald AG, Arora G, Ferrandino R, Gerace EL, Mohammednetej M, Nosair W, Rattila S, Subic AZ, Rolfes R 2016 Identification of genes in *Candida glabrata* conferring altered responses to caspofungin, a cell wall synthesis inhibitor. G3 (Bethesda) 6:2893–2907. doi:10.1534/g3.116.032490. 27449515 PMC5015946

[17] Bhakt P, Shivarathri R, Kumar Choudhary D, Borah S, Kaur R 2018 Fluconazole-induced actin cytoskeleton remodeling requires phosphatidylinositol 3-phosphate 5-kinase in the pathogenic yeast *Candida glabrata*. Mol Microbiol 110:425–443. doi:10.1111/mmi.14110. 30137648 PMC6221164

[18] Healey KR, Zhao Y, Perez WB, Lockhart SR, Sobel JD, Farmakiotis D, Kontoyiannis DP, Sanglard D, Taj-Aldeen SJ, Alexander BD, Jimenez-Ortigosa C, Shor E, Perlin DS 2016 Prevalent mutator genotype identified in fungal pathogen *Candida glabrata* promotes multidrug resistance. Nat Commun 7:11128. doi:10.1038/ncomms11128. 27020939 PMC5603725

[19] De Craene J-O, Bertazzi D, Bär S, Friant S, De Craene J-O, Bertazzi DL, Bär S, Friant S 2017 Phosphoinositides, major actors in membrane trafficking and lipid signaling pathways. IJMS 18:634. doi:10.3390/ijms18030634. PMC5372647 28294977

[20] Vernay A, Schaub S, Guillas I, Bassilana M, Arkowitz RA 2012 A steep phosphoinositide bis-phosphate gradient forms during fungal filamentous growth. J Cell Biol 198:711–730. doi:10.1083/jcb.201203099. 22891265 PMC3514036

[21] Ghugtyal V, Garcia-Rodas R, Seminara A, Schaub S, Bassilana M, Arkowitz RA 2015 Phosphatidylinositol-4-phosphate-dependent membrane traffic is critical for fungal filamentous growth. Proc Natl Acad Sci U S A 112:8644–8649. doi:10.1073/pnas.1504259112. 26124136 PMC4507248

[22] O’Meara TR, Veri AO, Polvi EJ, Li X, Valaei SF, Diezmann S, Cowen LE 2016 Mapping the Hsp90 genetic network reveals ergosterol biosynthesis and phosphatidylinositol-4-kinase signaling as core circuitry governing cellular stress. PLoS Genet 12:e1006142. doi:10.1371/journal.pgen.1006142. 27341673 PMC4920384

[23] Gary JD, Wurmser AE, Bonangelino CJ, Weisman LS, Emr SD 1998 Fablp is essential for PtdIns(3)P 5-kinase activity and the homeostasis of vacuolar size and membrane maintenance. J Cell Biol 143:65–79. doi:10.1083/jcb.143.1.65. 9763421 PMC2132800

[24] McCartney AJ, Zhang Y, Weisman LS 2014 Phosphatidylinositol 3,5-bisphosphate: low abundance, high significance. Bioessays 36:52–64. doi:10.1002/bies.201300012. 24323921 PMC3906640

[25] Strahl T, Thorner J 2007 Synthesis and function of membrane phosphoinositides in budding yeast, *Saccharomyces cerevisiae*. Biochim Biophys Acta 1771:353–404. doi:10.1016/j.bbalip.2007.01.015. 17382260 PMC1868553

[26] Ho CY, Alghamdi TA, Botelho RJ 2012 Phosphatidylinositol-3,5-bisphosphate: no longer the poor PIP2. Traffic 13:1–8. doi:10.1111/j.1600-0854.2011.01246.x. 21736686

[27] Gary JD, Weisman LS, Emr SD 2002 Regulation of Fab1 phosphatidylinositol 3-phosphate 5-kinase pathway by Vac7 protein and Fig4, a polyphosphoinositide phosphatase family member. Mol Biol Cell 13:2170–2179. doi:10.1091/mbc.01-10-0498. 11950935 PMC102265

[28] Bonangelino CJ, Nau JJ, Duex JE, Brinkman M, Wurmser AE, Gary JD, Emr SD, Weisman LS 2002 Osmotic stress-induced increase of phosphatidylinositol 3,5-bisphosphate requires Vac14p, an activator of the lipid kinase Fab1p. J Cell Biol 156:1015–1028. doi:10.1083/jcb.200201002. 11889142 PMC2173454

[29] Rudge SA, Anderson DM, Emr SD 2004 Vacuole size control: regulation of PtdIns(3,5)P_2_ levels by the vacuole-associated Vac14-Fig4 complex, a PtdIns(3,5)P_2_-specific phosphatase. Mol Biol Cell 15:24–36. doi:10.1091/mbc.e03-05-0297. 14528018 PMC307524

[30] Dove SK, Piper RC, McEwen RK, Yu JW, King MC, Hughes DC, Thuring J, Holmes AB, Cooke FT, Michell RH, Parker PJ, Lemmon MA 2004 Svp1p defines a family of phosphatidylinositol 3,5-bisphosphate effectors. EMBO J 23:1922–1933. doi:10.1038/sj.emboj.7600203. 15103325 PMC404323

[31] Jin N, Chow CY, Liu L, Zolov SN, Bronson R, Davisson M, Petersen JL, Zhang Y, Park S, Duex JE, Goldowitz D, Meisler MH, Weisman LS 2008 VAC14 nucleates a protein complex essential for the acute interconversion of PI3P and PI(3,5)P2 in yeast and mouse. EMBO J 27:3221–3234. doi:10.1038/emboj.2008.248. 19037259 PMC2600653

[32] Cota JM, Grabinski JL, Talbert RL, Burgess DS, Rogers PD, Edlind TD, Wiederhold NP 2008 Increases in SLT2 expression and chitin content are associated with incomplete killing of *Candida glabrata* by caspofungin. Antimicrob Agents Chemother 52:1144–1146. doi:10.1128/AAC.01542-07. 18086838 PMC2258485

[33] Arana DM, Prieto D, Román E, Nombela C, Alonso-Monge R, Pla J 2009 The role of the cell wall in fungal pathogenesis. Microb Biotechnol 2:308–320. doi:10.1111/j.1751-7915.2008.00070.x. 21261926 PMC3815752

[34] Patenaude C, Zhang Y, Cormack B, Köhler J, Rao R 2013 Essential role for vacuolar acidification in *Candida albicans* virulence. J Biol Chem 288:26256–26264. doi:10.1074/jbc.M113.494815. 23884420 PMC3764829

[35] Rai MN, Sharma V, Balusu S, Kaur R 2015 An essential role for phosphatidylinositol 3-kinase in the inhibition of phagosomal maturation, intracellular survival and virulence in *Candida glabrata*. Cell Microbiol 17:269–287. doi:10.1111/cmi.12364. 25223215

[36] Efe JA, Botelho RJ, Emr SD 2007 Atg18 regulates organelle morphology and Fab1 kinase activity independent of its membrane recruitment by phosphatidylinositol 3,5-bisphosphate. Mol Biol Cell 18:4232–4244. doi:10.1091/mbc.e07-04-0301. 17699591 PMC2043547

