# Supplementary material for: Essential Role for the Phosphatidylinositol 3,5-Bisphosphate Synthesis Complex in Caspofungin Tolerance and Virulence in Candida glabrata
Source: Antimicrob Agents Chemother. 2019 Jul 25;63(8):e00886-19. doi: 10.1128/AAC.00886-19 (PMC6658794; doi:10.1128/AAC.00886-19)
Supplement: Supplemental file 1 [file AAC.00886-19-s0001.pdf]

**Supplementary Information: Two tables (S1 and S2) and seven figures (S1-S7)**

1. **Supplementary tables**

**Supplementary table S1: List of strains and plasmids used in the study**

**Supplementary table S2: List of primers used in the study**

2. **Supplementary figures**

**Supplementary figures S1 – S7**

**Figure S1: The *Cgfig4Δ* mutant contain regular-sized vacuoles. (A)** Schematic depiction of the domain structure of CgFab1, CgFig4, CgVac7, CgVac14 and CgAtg18 proteins as determined using the NCBI Conserved domain tool (www.ncbi.nlm.nih.gov/Structure/cdd/wrpsb.cgi) in combination with the SMART tool (http://smart.embl-heidelberg.de). Amino acid positions of predicted domains are indicated. CgFab1 domains (FYVE, TCP1 and PIP5 kinase) are represented as previously described in Bhakt *et al.* 2018. In addition to the phohphoinositide polyphosphatase domain, CgFig4 contains SacI homology domain which is conserved in several lipid phosphatases. CgVac7 contains two vacuolar segregation domains (Vac7) at 320-349 aa and 851-934 aa positions. The second domain was predicted by the SMART tool to contain a transmembrane domain. CgVac14 contains Fab1-binding region and Fig4-binding region at the N- and C- termini, respectively. CgVac14 also contains tandem HEAT repeats (indicated with two-directional arrows), which are known to be present in proteins with scaffolding functions. CgAtg18 contains WD40-repeats, which represent inter molecular interaction sites **(B)** Representative confocal microscopy images of FM4-64 stained, YPD-grown log phase cultures of indicated *C. glabrata* strains. DIC, Differential interference contrast; Bar = 1.0 μm.

**Figure S2: The *CgFIG4* deletion could not rescue the large vacuole phenotype of the *Cgvac14Δ* mutant.** Representative confocal microscopy images of FM4-64 stained, YPD-grown log phase cultures of indicated *C. glabrata* strains. Images were captured using the Leica confocal microscope. DIC, Differential interference contrast; Bar = 2.0 µm.

**Figure S3: Overexpression of PI(3,5)P2 regulatory complex constituents, *CgFAB1, CgFIG4, CgVAC7* and *CgVAC14*, have no effect on the growth of *wild-type* and *Cgfig4Δ* strains. (A)** Serial dilution spot analysis to assess the susceptibility of indicated *C. glabrata* strains towards fluconazole (FLC; 16 µg/ml), congo red (CR, 2 mg/ml), calcofluor white (CFW; 2 mg/ml) and zinc chloride ($ZnCl_2$; 8 mM). **(B)** Liquid growth assay-based analysis of indicated *C. glabrata* strains in the medium lacking (CAA) or containing caspofungin (CSP; 75 ng/ml).

**Figure S4: The constitutively active CgFab1 enzyme reverses the large vacuole phenotype of *Cgvac7Δ* and *Cgvac14Δ* mutants. (A)** Amino acid sequence alignment of the C-terminus kinase domain of *C. glabrata* and *S. cerevisiae*. The conserved threonine at 2076 position in CgFab1, which was mutated to alanine, is marked. **(B)** Representative confocal microscopy images of FM4-64 stained, YPD-grown log phase cultures of indicated *C. glabrata* strains. Images were captured using the Leica confocal microscope. DIC, Differential interference contrast; Bar = 2.0 µm.

**Figure S5: Deletion of PI3K subunits had no effect on caspofungin susceptibility of *C. glabrata*.** Liquid growth assay-based analysis of *wt*, *Cgvps15Δ* and *Cgvps34Δ* mutants in the medium lacking (CAA) or containing caspofungin (CSP; 75 ng/ml).

**Figure S6: Deletion of *CgATG18* had no effect on stress susceptibility and vacuolar morphology in *C. glabrata*. (A)** Representative confocal microscopy images of FM4-64 stained, YPD-grown log phase cultures of *wt* and *Cgatg18Δ* strains. DIC, Differential interference contrast; Bar = 1.0 µm. **(B)** Serial dilution spot analysis to assess the susceptibility of *wt* and *Cgatg18Δ* strains towards fluconazole (FLC; 16 µg/ml), congo red (CR, 2 mg/ml), calcofluor white (CFW; 2 mg/ml) and zinc chloride ($ZnCl_2$; 8 mM). **(C)** Liquid growth assay-based analysis of *wt* and *Cgatg18Δ* mutant in the medium lacking (CAA) or containing caspofungin (CSP; 75 ng/ml).

**Figure S7: The fungal Vac7 protein shows three weakly conserved regions.** Multiple amino acid sequence alignment of Vac7 of *Aspergillus fumigatus*, *C. albicans*, *C. glabrata*, *C. parapsilosis*, *C. tropicalis*, *Histoplasma capsulatum* and *S. cerevisiae*. The conserved regions are marked.

**Table S1: List of strains and plasmids used in the study.**

| Yeast strain | Genotype | Reference |
|---|---|---|
| BG2 | Clinical isolate | (1) |
| YRK19 | *ura3Δ::Tn903 G418R* (BG14) | (2) |
| YRK20 | *URA3* (BG462) | (3) |
| YRK695 | *URA3 Cgvps15Δ::nat1* | (4) |
| YRK710 | *URA3 Cgvps34Δ::nat1* | (4) |
| YRK1070 | *ura3Δ::Tn903 G418R Cgfab1Δ::nat1* | (5) |
| YRK1084 | *ura3Δ::Tn903 G418R* (YRK19)*/pRK74* | (5) |
| YRK1138 | *ura3Δ::Tn903 G418R Cgfab1Δ::nat1/pRK74* | (5) |
| YRK1139 | *ura3Δ::Tn903 G418R Cgfab1Δ::nat1/pRK1033* | (5) |
| YRK1287 | *URA3 Cgfab1Δ::nat1* | (5) |
| YRK1597 | *URA3 Cgfig4Δ::nat1* | This study |
| YRK1601 | *URA3 Cgvac14Δ::nat1* | (5) |
| YRK1603 | *ura3Δ::Tn903 G418R Cgvac14Δ::nat1* | This study |
| YRK1692 | *URA3 Cgvac7Δ::nat1* | (5) |
| YRK1694 | *ura3Δ::Tn903 G418R Cgvac7Δ::nat1* | This study |
| YRK1766 | *ura3Δ::Tn903 G418R* (YRK19)*/pRK1106* | (5) |
| YRK1771 | *ura3Δ::Tn903 G418R Cgvac7Δ::nat1/pRK1106* | This study |
| YRK1777 | *ura3Δ::Tn903 G418R Cgvac14Δ::nat1/pRK1106* | This study |
| YRK2027 | *ura3Δ::Tn903 G418R Cgfig4Δ::nat1* | This study |
| YRK2059 | *ura3Δ::Tn903 G418R Cgfig4Δ::nat1/pRK1106* | This study |
| YRK2588 | *ura3Δ::Tn903 G418R Cgvac14Δ::nat1/pRK1638* | This study |
| YRK2591 | *ura3Δ::Tn903 G418R Cgfig4Δ::nat1/pRK1635* | This study |
| YRK2595 | *ura3Δ::Tn903 G418R Cgfig4Δ::nat1/pRK74* | This study |
| YRK2597 | *ura3Δ::Tn903 G418R Cgvac7Δ::nat1/pRK74* | This study |
| YRK2599 | *ura3Δ::Tn903 G418R Cgvac14Δ::nat1/pRK74* | This study |
| YRK2609 | *ura3Δ::Tn903 G418R Cgvac7Δ::nat1/pRK1653* | This study |
| YRK2678 | *ura3Δ::Tn903 G418R Cgvac7Δfig4Δ::nat1* | This study |
| YRK2765 | *URA3 Cgvac7Δfig4Δ::nat1* | This study |
| YRK2751 | *ura3Δ::Tn903 G418R* (YRK19)*/pRK1680* | This study |
| YRK2755 | *ura3Δ::Tn903 G418R Cgvac7Δ::nat1/pRK1680* | This study |
| YRK2757 | *ura3Δ::Tn903 G418R Cgvac14Δ::nat1/pRK1680* | This study |
| YRK2790 | *ura3Δ::Tn903 G418R Cgvac7Δfig4Δ::nat1/pRK1696* | This study |
| YRK2850 | *ura3Δ::Tn903 G418R Cgfig4Δ::nat1/pRK1680* | This study |
| YRK2852 | *ura3Δ::Tn903 G418R Cgfab1Δ::nat1/pRK1680* | This study |
| YRK2855 | *ura3Δ::Tn903 G418R Cgvac7Δfig4Δ::nat1/pRK1680* | This study |
| YRK2879 | *ura3Δ::Tn903 G418R* (YRK19)*/pRK1738* | This study |

| | | |
|---|---|---|
| YRK2883 | *ura3Δ::Tn903 G418R Cgfig4Δ::nat1/pRK1738* | This study |
| YRK2885 | *ura3Δ::Tn903 G418R Cgfab1Δ::nat1/pRK1738* | This study |
| YRK2887 | *ura3Δ::Tn903 G418R Cgvac7Δ::nat1/pRK1738* | This study |
| YRK2889 | *ura3Δ::Tn903 G418R Cgvac14Δ::nat1/pRK1738* | This study |
| YRK2935 | *ura3Δ::Tn903 G418R Cgfab1Δfig4Δ::nat1* | This study |
| YRK2937 | *ura3Δ::Tn903 G418R Cgvac14Δfig4Δ::nat1* | This study |
| YRK2982 | *ura3Δ::Tn903 G418R Cgfab1Δfig4Δ::nat1/pRK999* | This study |
| YRK2984 | *ura3Δ::Tn903 G418R Cgvac14Δfig4Δ::nat1/pRK999* | This study |
| YRK2986 | *ura3Δ::Tn903 G418R Cgfab1Δfig4Δ::nat1/pRK1696* | This study |
| YRK2988 | *ura3Δ::Tn903 G418R Cgvac14Δfig4Δ::nat1/pRK1696* | This study |
| YRK2990 | *ura3Δ::Tn903 G418R Cgvac14Δfig4Δ::nat1/pRK1738* | This study |
| YRK2994 | *ura3Δ::Tn903 G418R* (YRK19)*/pRK999* | This study |
| YRK2995 | *ura3Δ::Tn903 G418R Cgfig4Δ::nat1/pRK999* | This study |
| YRK2997 | *ura3Δ::Tn903 G418R Cgfab1Δ::nat1/pRK999* | This study |
| YRK2998 | *ura3Δ::Tn903 G418R Cgvac7Δ::nat1/pRK999* | This study |
| YRK2999 | *ura3Δ::Tn903 G418R Cgvac14Δ::nat1/pRK999* | This study |
| YRK3009 | *URA3 Cgfab1Δfig4Δ::nat1* | This study |
| YRK3011 | *URA3 Cgvac14Δfig4Δ::nat1* | This study |
| YRK3019 | *ura3Δ::Tn903 G418R* (YRK19)*/pRK1696* | This study |
| YRK3020 | *ura3Δ::Tn903 G418R Cgfab1Δ::nat1/pRK1696* | This study |
| YRK3021 | *ura3Δ::Tn903 G418R Cgvac7Δ::nat1/pRK1696* | This study |
| YRK3022 | *ura3Δ::Tn903 G418R Cgvac14Δ::nat1/pRK1696* | This study |
| YRK3023 | *ura3Δ::Tn903 G418R Cgfig4Δ::nat1/pRK1696* | This study |
| YRK3029 | *ura3Δ::Tn903 G418R Cgfig4Δ::nat1/pRK1780* | This study |
| YRK3031 | *ura3Δ::Tn903 G418R Cgvac7Δ::nat1/pRK1780* | This study |
| YRK3033 | *ura3Δ::Tn903 G418R Cgvac14Δ::nat1/pRK1780* | This study |
| YRK3059 | *ura3Δ::Tn903 G418R* (YRK19)*/pRK1791* | This study |
| YRK3063 | *ura3Δ::Tn903 G418R Cgfab1Δ::nat1/pRK1791* | This study |
| YRK3065 | *ura3Δ::Tn903 G418R Cgfig4Δ::nat1/pRK1791* | This study |
| YRK3067 | *ura3Δ::Tn903 G418R Cgvac7Δ::nat1/pRK1791* | This study |
| YRK3069 | *ura3Δ::Tn903 G418R Cgvac14Δ::nat1/pRK1791* | This study |
| YRK3094 | *ura3Δ::Tn903 G418R* (YRK19)*/pRK1780* | This study |
| YRK3095 | *ura3Δ::Tn903 G418R Cgfab1Δ::nat1/pRK1780* | This study |
| YRK3102 | *ura3Δ::Tn903 G418R Cgvac7Δfig4Δ::nat1/pRK999* | This study |
| YRK3104 | *ura3Δ::Tn903 G418R Cgfab1Δfig4Δ::nat1/pRK1791* | This study |

| Plasmid | Description | Reference |
|---|---|---|
| pRK74 | A CEN-ARS plasmid (pGRB2.2) of *C. glabrata* carrying *S. cerevisiae URA3* as a selection marker. MCS sites are flanked by *S. cerevisiae PGK1* promoter at one end and by 3′ UTR of *HIS3* at the other end. | (6) |
| pRK999 | pCU-PDC1 plasmid | Addgene (Plasmid #45323) |
| pRK1000 | pCU-PDC1-GFP plasmid | Addgene (Plasmid #45324) |
| pRK1018 | pGRB2.3 plasmid | Addgene (Plasmid #45343) |
| pRK1033 | *CgFAB1* (6.31 kb) cloned in BamH*I*-Sal*I* sites of pRK74 plasmid | (5) |
| pRK1106 | *CgFAB1* (6.31 kb) cloned in BamH*I*-Xma*I* sites of pGRB2.3 plasmid (pRK1018) | (5) |
| pRK1144 | *CgFAB1*$^{GKSG\rightarrow VKSV}$ cloned in the pRK74 plasmid | (5) |
| pRK1345 | *CgFAB1* (6.31 kb) cloned in BamH*I*-Xma*I* sites of pRK74 plasmid with SFB tag at the C-terminus | This study |
| pRK1635 | *CgFIG4* (2.61 kb) cloned in Spe*I*-BamH*I* sites of pRK74 plasmid | This study |
| pRK1638 | *CgVAC14* (2.6 kb) cloned in Spe*I*-EcoR*I* sites of pRK74 plasmid | This study |
| pRK1653 | *CgVAC7* (3.23 kb) cloned in EcoR*I*-Xma*I* sites of pRK74 plasmid | This study |
| pRK1680 | *CgVAC7* (3.23 kb) cloned in Xma*I*-EcoR*I* sites of pRK1000 plasmid | This study |
| pRK1696 | *CgFIG4* (2.61 kb) cloned in Spe*I*-Xma*I* sites of pRK1000 plasmid | This study |
| pRK1738 | *CgVAC14* (2.6 kb) cloned in Spe*I*-EcoR*I* sites of pRK1000 plasmid | This study |
| pRK1780 | *CgFAB1*$^{T2076A}$ cloned in the pRK1345 plasmid | This study |
| pRK1791 | *CgFAB1* (6.31 kb) cloned in Xma*I*-Sal*I* sites of pRK999 plasmid | This study |

## References


1. Fidel PL, Cutright JL, Tait L, Sobel JD. 1996. A murine model of *Candida glabrata* vaginitis. J Infect Dis **173**:425–431.

2. Cormack BP, Falkow S. 1999. Efficient homologous and illegitimate recombination in the opportunistic yeast pathogen *Candida glabrata*. Genetics **151**:979–987.

3. Orta-Zavalza E, Guerrero-Serrano G, Gutierrez-Escobedo G, Canas-Villamar I, Juarez-Cepeda J, Castano I, De Las Penas A. 2013. Local silencing controls the oxidative stress response and the multidrug resistance in *Candida glabrata*. Mol Microbiol **88**:1135–1148.

4. Rai MN, Sharma V, Balusu S, Kaur R. 2015. An essential role for phosphatidylinositol 3-kinase in the inhibition of phagosomal maturation, intracellular survival and virulence in *Candida glabrata*. Cell Microbiol **17**:269–287.

5. Bhakt P, Shivarathri R, Kumar Choudhary D, Borah S, Kaur R. 2018. Fluconazole-induced actin cytoskeleton remodeling requires phosphatidylinositol 3-phosphate 5-kinase in the pathogenic yeast *Candida glabrata*. Mol Microbiol **110**:425–443.

6. Frieman MB, McCaffery JM, Cormack BP. 2002. Modular domain structure in the *Candida glabrata* adhesin Epa1p, a beta 1,6 glucan-cross-linked cell wall protein. Mol Microbiol **46**:479–492.

**Table S2: List of primers used in the study**

| **Primer** | **Sequence (5'→ 3')** | **Description** |
|---|---|---|
| **For generation of deletion strains** | | |
| OgRK2139 | CGCCAAGAGACAGCCACCTACAATG | *CgFIG4* 5'UTR Forward |
| OgRK2140 | GCGTCGACCTGCAGCGTACGCTAATAGTCCACGTCCCCAG | *CgFIG4* 5'UTR Reverse |
| OgRK2141 | CGACGGTGTCGGTCTCGTAGGTGGTTCCAGTTATAGTTTTG | *CgFIG4* 3'UTR Forward |
| OgRK2142 | TCAAACTGCTGTTACCATCATTGC | *CgFIG4* 3'UTR Reverse |
| OgRK2143 | AAACACCGGAGAGAAGCTCACC | *CgFIG4* Internal check Forward |
| OgRK2144 | TACACGGATTTCCCCAGAACAG | *CgFIG4* Internal check Reverse |
| OgRK2145 | CATAGTGATCTCGTCAACTCTAG | *CgFIG4* 5' Integration check Forward |
| OgRK2146 | CCTCCTGATTATTCACTGCATG | *CgFIG4* 3' Integration check Reverse |
| **For gene cloning** | | |
| OgRK1178 | CGCGTCGACTTAGTTTGTATCTTGATACCA | *CgFAB1* Cloning Reverse |
| OgRK2298 | CATAGAATTCATGGACGATGAGAAGAAGC | *CgVAC7* Cloning Forward |
| OgRK2299 | CATACTCGAGTTACTGTTTACTTGGACG | *CgVAC7* Cloning Reverse |
| OgRK2300 | CATACCCGGGATGGACGATGAGAAGAAGC | *CgVAC7* Cloning Forward |
| OgRK2301 | CATAGAATTCCTGTTTACTTGGACG | *CgVAC7* Cloning Reverse |
| OgRK2306 | CATAACTAGTATGGACAAATCAATTGAG | *CgVAC14* Cloning Forward |
| OgRK2307 | CATAGAATTCTTAAGTAAACTTTTTTAGTTTG | *CgVAC14* Cloning Reverse |
| OgRK2308 | CATAGAATTCAGTAAACTTTTTTAGTTTGC | *CgVAC14* Cloning Reverse |
| OgRK2309 | CATAACTAGTATGGCTGGTGGATATGAGTC | *CgFIG4* Cloning Forward |
| OgRK2310 | CATAGGATCCTTACATAATAGGCTCCCAGC | *CgFIG4* Cloning Reverse |
| OgRK2311 | CATAGGATCCCATAATAGGCTCCCAGC | *CgFIG4* Cloning Reverse |
| OGRK3429 | CAGACCCGGGATGGCTATTGGTCAAGGGACGCTTC | *CgFAB1* Cloning Forward |
| **For site-directed mutagenesis** | | |
| OgRK2009 | CAGAGTCGACGGGCTCCCGCTGGCCCTGAGGGT | SFB tag reverse primer used for amplifying 3' half of *CgFAB1-SFB* |
| OgRK2025 | GTCGTCCGATTAGAATATTCTG | *CgFAB1* internal forward primer used for amplifying 5' half of *CgFAB1-SFB* |
| OgRK3403 | GCAACCAACAGTGGTAGCTCCCAAACAGTATAAG | *CgFAB1*-SDM $^{T2076A}$ Forward |
| OgRK3404 | CTTATACTGTTTGGGAGCTACCACTGTTGGTTGC | *CgFAB1*-SDM $^{T2076A}$ Reverse |

# Figure S1

A

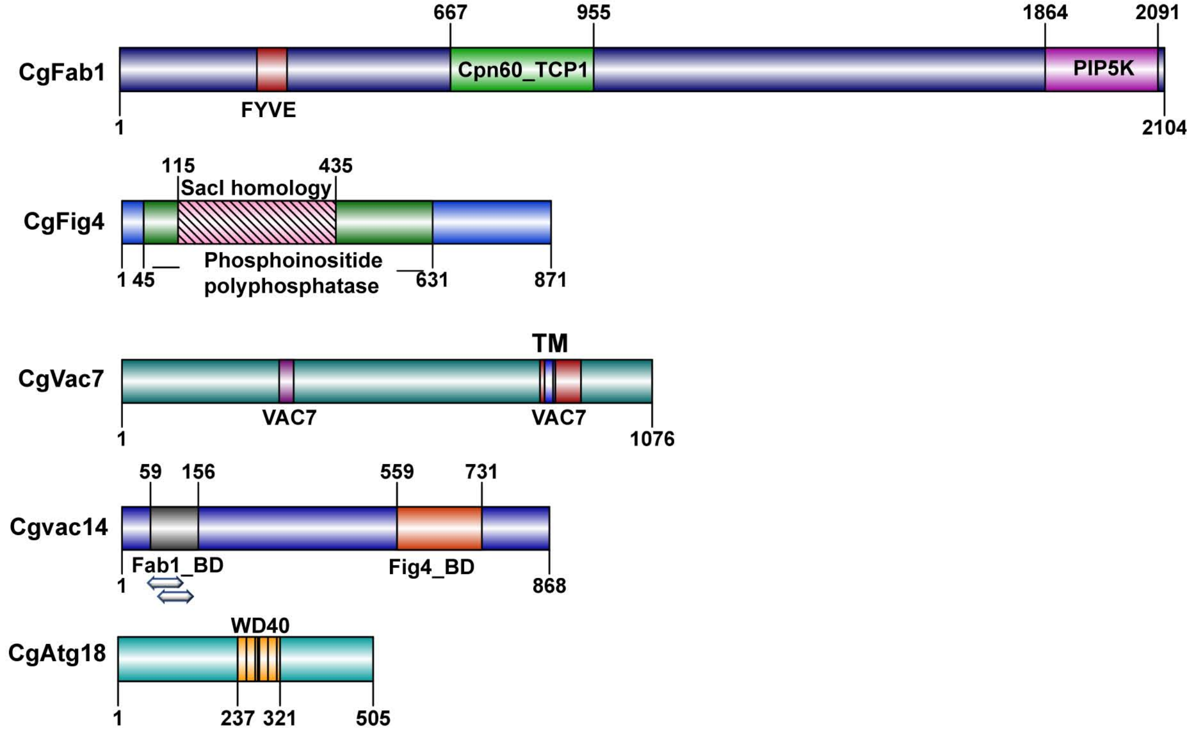

667
955
1864
2091
CgFab1
Cpn60_TCP1
PIP5K
FYVE
1
2104
115
435
SacI homology
CgFig4
Phosphoinositide
polyphosphatase
1
45
631
871
TM
CgVac7
VAC7
VAC7
1
1076
59
156
559
731
Cgvac14
Fab1_BD
Fig4_BD
1
868
WD40
CgAtg18
1
237
321
505


B

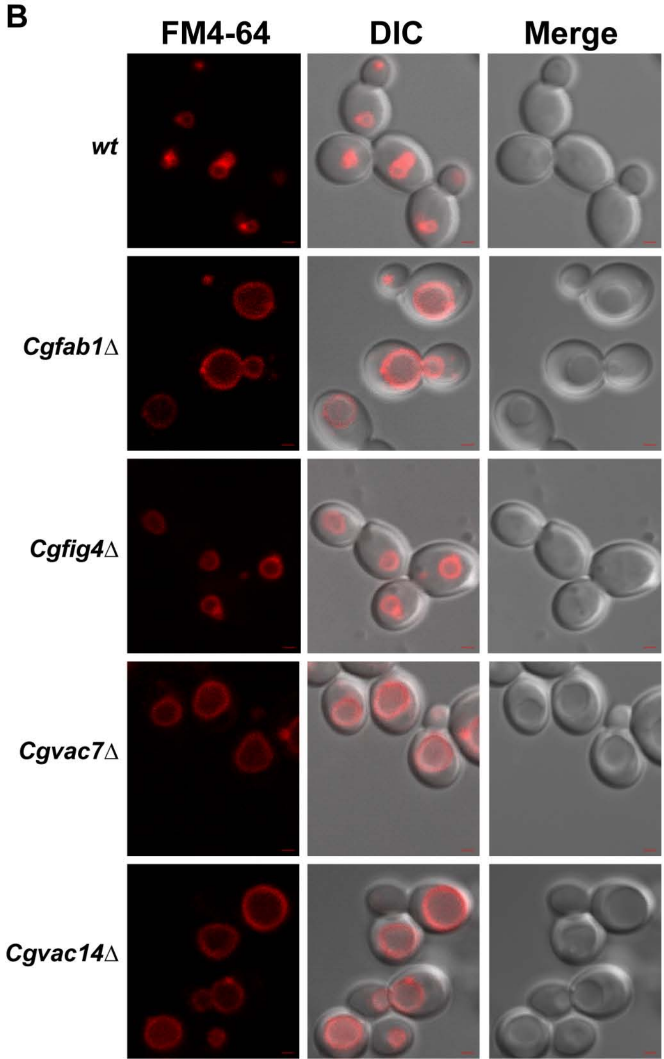

FM4-64
DIC
Merge
wt
Cgfab1Δ
Cgfig4Δ
Cgvac7Δ
Cgvac14Δ

# Figure S2

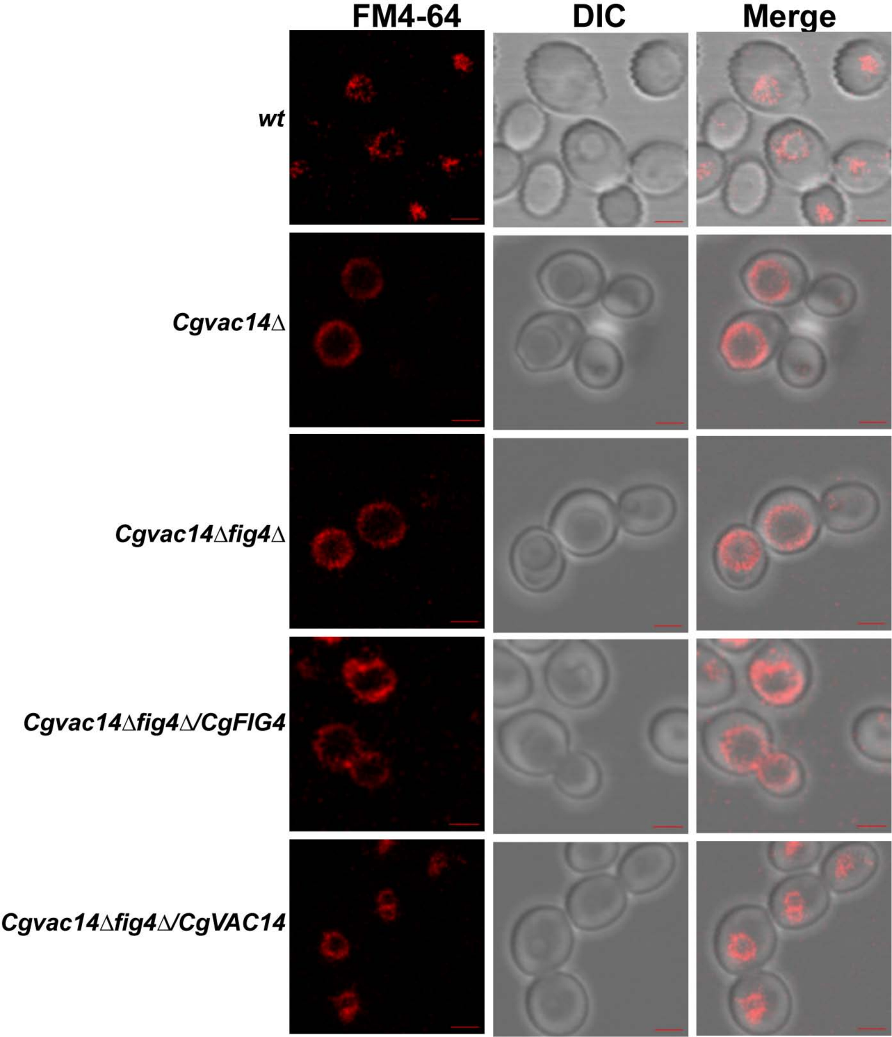
FM4-64
DIC
Merge
wt
Cgvac14Δ
Cgvac14Δfig4Δ
Cgvac14Δfig4Δ/CgFIG4
Cgvac14Δfig4Δ/CgVAC14

# Figure S3

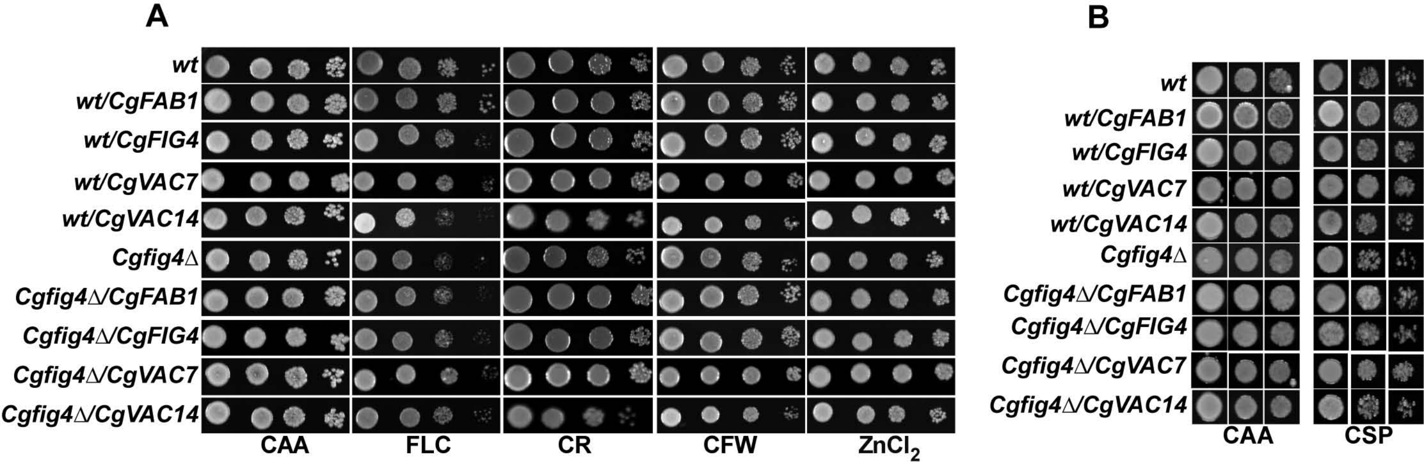
A
wt
wt/CgFAB1
wt/CgFIG4
wt/CgVAC7
wt/CgVAC14
Cgfig4Δ
Cgfig4Δ/CgFAB1
Cgfig4Δ/CgFIG4
Cgfig4Δ/CgVAC7
Cgfig4Δ/CgVAC14
CAA
FLC
CR
CFW
ZnCl2
B
wt
wt/CgFAB1
wt/CgFIG4
wt/CgVAC7
wt/CgVAC14
Cgfig4Δ
Cgfig4Δ/CgFAB1
Cgfig4Δ/CgFIG4
Cgfig4Δ/CgVAC7
Cgfig4Δ/CgVAC14
CAA
CSP

# Figure S4

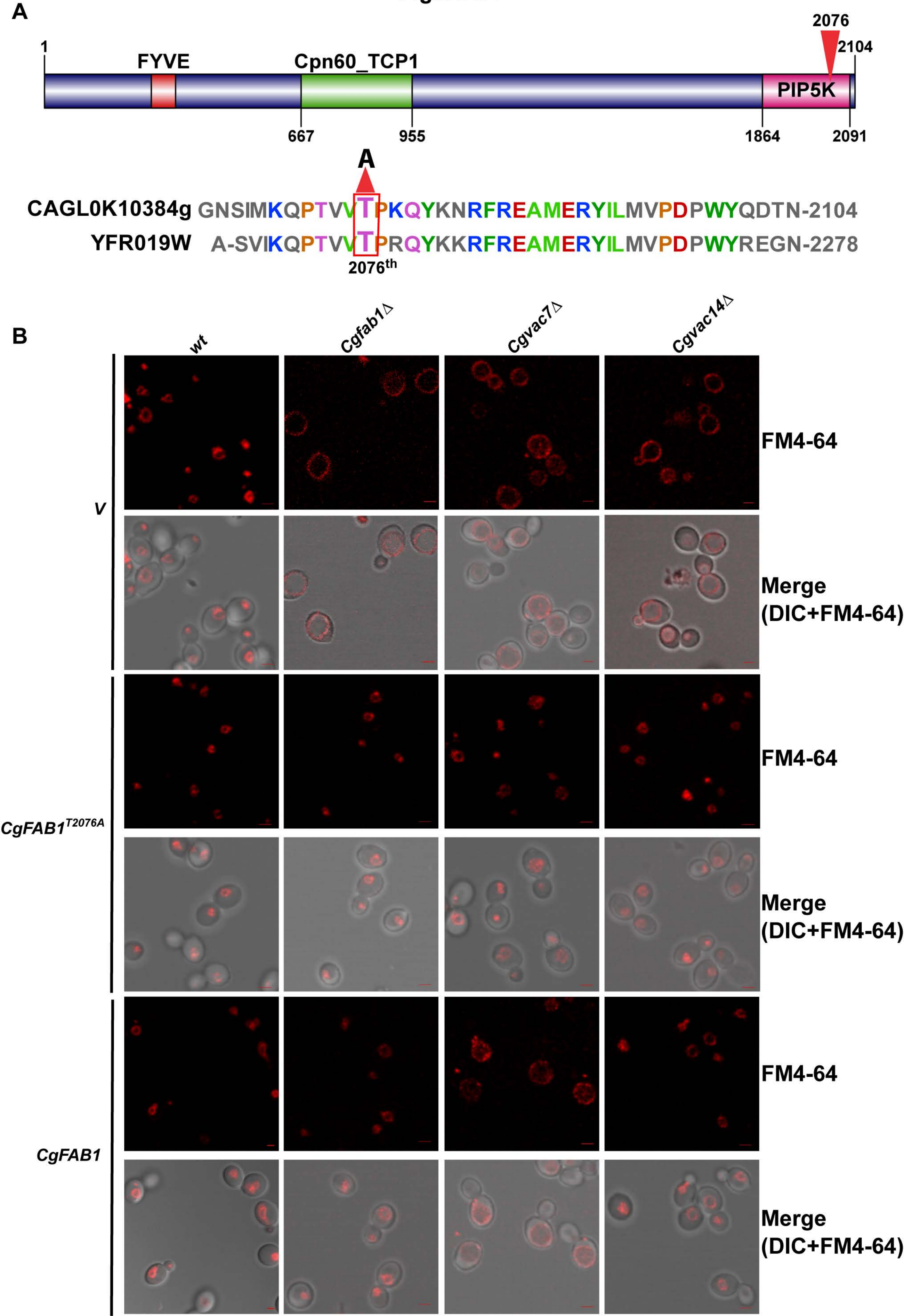

A
1
FYVE
Cpn60_TCP1
2076
2104
PIP5K
667
955
1864
2091
A
CAGL0K10384g GNSIMKQPTVVTPKQYKNRFREAMERYILMVPDPWYQDTN-2104
YFR019W A-SVIKQPTVVTPRQYKKRFREAMERYILMVPDPWYREGN-2278
2076th
B
wt
Cgfab1Δ
Cgvac7Δ
Cgvac14Δ
V
FM4-64
Merge (DIC+FM4-64)
CgFAB1T2076A
FM4-64
Merge (DIC+FM4-64)
CgFAB1
FM4-64
Merge (DIC+FM4-64)

# Figure S5

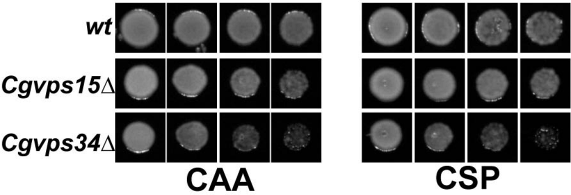

# Figure S6

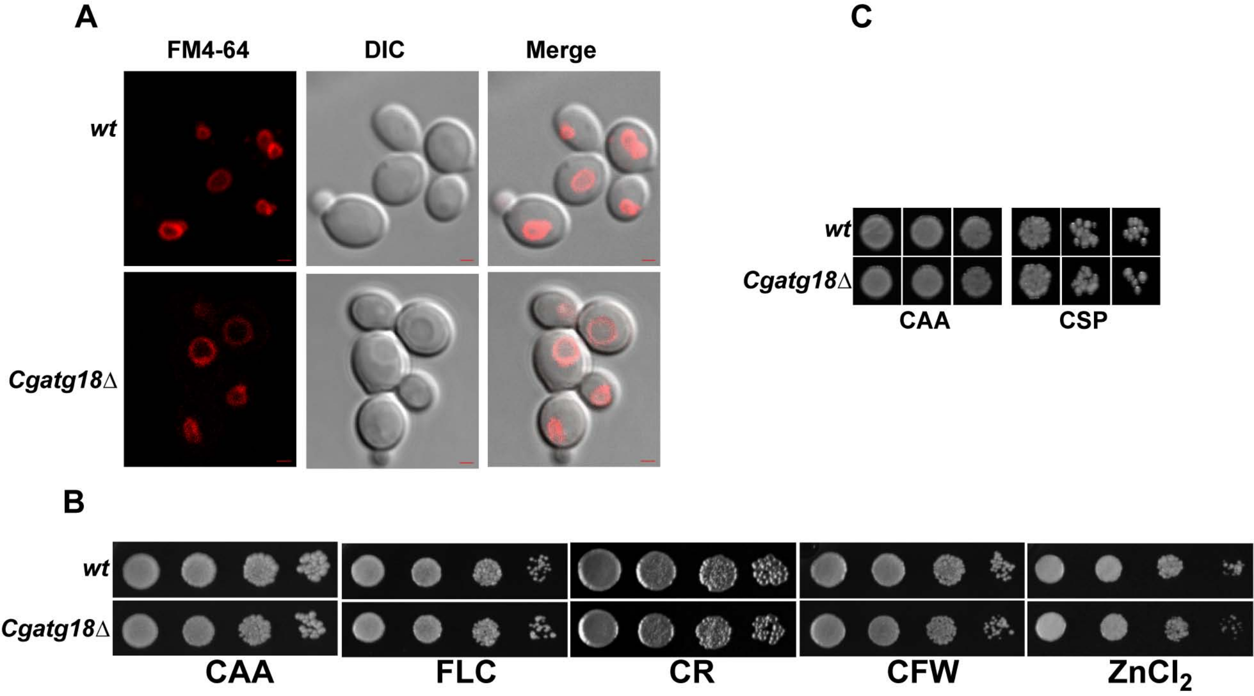
A
FM4-64
DIC
Merge
wt
Cgatg18Δ
B
wt
Cgatg18Δ
CAA
FLC
CR
CFW
ZnCl2
C
wt
Cgatg18Δ
CAA
CSP

# Figure S7

```
A. fumigatus (Q4X210)     RKEKRK-TRKPAALASGAASSKADIFEAKVASAVDEADVSDSDETFVYESNPPDPYPVRQ 415
H. capsulatum (A6RCP0)    KKEKKKPVRRPTALPAGTVSSKADIFEAKVASAVDEADSSDSAETFIYESNPPDSRPAHP 106
C. parapsilosis (G8B9E6)  DA-DTSNKQVKQKKVAKQSSTKTDFFAAKLASAVDDVESSDSDETFVYENNDDTFDTTNN 349
C. albicans (Q59PA6)      ENTDAPPKQQTKKKISKQNSTKTDFFAARLASAVDDIESSDSDETFIYENNDTELDDNAS 78
C. tropicalis (C5MCH8)    DTLDSQS-KPNKKKISKQTSTKTDFFAARLASAVDDIESSDSDETFVYENNGNGFDTNTN 416
C. glabrata (Q6FTM4)      DEI----TTQTTESSNAHRPTKTDFFAARLASAVGENEVSDSEETFVYESAANSTKNMIY 359
S. cerevisiae (P53950)    NNA----INDDSHESNSEKPTKADFFAARLATAVGENEISDSEETFVYESAANSTKNLIF 463
                                              :*:*:* *::*:**.: : *** ***:**.

A. fumigatus (Q4X210)     LAIGGATSFIIGITKPLVDVQVLAIQNVLASEQEIMLDLRVQAVNPNLFSVAVDDMDVNF 685
H. capsulatum (A6RCP0)    LLAGGATTAVVGLMKPLTGVHVKKIRNVLASEQQIMFGLEVQAINSNLMTLTVNNMDVNI 362
C. parapsilosis (G8B9E6)  LTVGFILGFVMATTKDLTGVSINSIENPIVSKDELVFNVVVEAFNPGWFSVDINEVELDL 808
C. albicans (Q59PA6)      LTIGFVLGFVLATTKDLTDVGITSIENPIVSKDELVFNVVIEAFNPGWFSVDINEVELDL 620
C. tropicalis (C5MCH8)    LVVGFMFGFILATTKDLTDVGITSIENPIVSKDELVFNIVVEAFNPGWFSVDINEVELDL 921
C. glabrata (Q6FTM4)      VSIGFVLGFLLAANKELREFDIVLMDNVISSTDELLFDLTTTAFNPGIFPIYVDEVEFDI 927
S. cerevisiae (P53950)    LMTGFILGFLLATNKELQDVDVVVMDNVISSSDELIFDITVSAFNPGFFSISVSQVDLDI 990
                          :  *     ::.  * *  . :  : * : * :::::.:   *.* . : : :.:::.::

A. fumigatus (Q4X210)     ----------EEGGTERWERVLQHPFELIVRGVIKYQLPLSSRYYSSPVSSSV------K 849
H. capsulatum (A6RCP0)    ----------EEGGTARWERVLQNPFELIVRGVVKYQLPLTSRMRSASINSQI------T 524
C. parapsilosis (G8B9E6)  ----QSHNGTDSDNTEKWEIISENPFDLIISGVLKYDLPLVSSTKSVVVRKIGYIDPTLF 933
C. albicans (Q59PA6)      KESTTSNDTNDNDNSKKWEIISSNPFDLIITGVLKYDLPFSRTSRSVVVRKTGYIDPTLF 768
C. tropicalis (C5MCH8)    ----KNKNNESDDNSKKWEIICANPFDLIITGILKYDLPFARTTRSVVVRKTGYIDPTLF 1054
C. glabrata (Q6FTM4)      YG-LDKRDSDESDDVTKWKLVIKHDYELILRGNIKYKVPFFNTARSIAVQKSAEVRPSKQ 1076
S. cerevisiae (P53950)    YKSKPNARDDKEDDTKKWKLLIKHDYELIVRGSMKYEVPFFNTQKSTAIQKDSMVHPGKK 1156
                                    ....  :*: :  : ::**: * :**.:*:     *  : .
```
